# Ethnobotanical Uses and Phytochemical, Pharmacological, Behavioral, and Toxicological Effects of *Montanoa* Species Used by Pre-Columbian Mesoamerican Cultures for Anxiety and Depression

**DOI:** 10.3390/molecules31172972

**Published:** 2026-08-25

**Authors:** Juan Francisco Rodríguez-Landa, María de Jesús Rovirosa-Hernández, Francisco García-Orduña, Gabriel Guillén-Ruiz, Jonathan Cueto-Escobedo, Frank Pulido-Criollo, Oscar Jerónimo Olmos-Vázquez, Ana Karen Limón-Vázquez

**Affiliations:** 1 Laboratorio de Neurofarmacología, Instituto de Neuroetología, Universidad Veracruzana, Xalapa 91190, VER, Mexico; analimon@uv.mx; 2 Instituto de Neuroetología, Universidad Veracruzana, Xalapa 91190, VER, Mexico; jrovirosa@uv.mx (M.d.J.R.-H.); fragarcia@uv.mx (F.G.-O.); 3 Investigadores por México-SECIHTI, Instituto de Neuroetología, Universidad Veracruzana, Xalapa 91190, VER, Mexico; gguillen@uv.mx; 4 Departamento de Investigación Clínica y Traslacional, Instituto de Ciencias de la Salud, Universidad Veracruzana, Xalapa 91190, VER, Mexico; jcueto@uv.mx; 5 Programa de Licenciatura en Enfermería, Universidad del Papaloapan (UNPA), Tuxtepec 68301, OAX, Mexico; fpcriollo@comunidad.unam.mx; 6 Servicios de Salud del Instituto Mexicano del Seguro Social (IMSS-Bienestar), Hospital General de Boca del Río, Servicio de Transfusión Sanguínea, Boca del Río 94290, VER, Mexico; oscarplsstahp1@gmail.com

**Keywords:** anxiety, bioactive phytochemical, depression, cihuapahtli, drug discovery, pre-Columbian Mesoamerican medicine

## Abstract

Ancient cultures have long studied plants for medicinal use. Today, multidisciplinary research focuses on re-evaluating these plants to discover drugs for treating various diseases, including psychiatric disorders. Pre-Columbian Mesoamerican cultures described three plants (*Montanoa tomentosa*, *Montanoa frutescens*, and *Montanoa grandiflora*) for treating both anxiety and depression disorders, which were called cihuapahtli in Nahuatl. This narrative review describes and analyzes the ethnobotanical use and phytochemical, pharmacological, behavioral, and toxicological effects of cihuapahtli, which can contribute to the discovery of anxiolytic and antidepressant drugs. The results show a significant advance in identifying bioactive phytochemicals, including triterpenes, diterpenes, monoterpenes, and sesquiterpenes, as well as sterols, flavonoids, and alkaloids. In particular, the mechanism of action underlying their anxiolytic and antidepressant effects involves the activation of oxytocinergic neurons in the paraventricular nucleus of the hypothalamus, as well as that of the GABAergic system by targeting GABA_A_ receptors. The three *Montanoa* species have the potential to help us develop anxiolytic and antidepressant drugs; however, future studies are still required to evaluate toxicity, dose standardization, possible variability in plant-derived compounds, pharmacokinetic parameters, and pharmacological interactions. Only then will preclinical evidence be sufficiently robust to justify translational studies evaluating the safety and efficacy of these plant extracts for the development of anxiolytic and antidepressant phytomedicines.

## 1. Introduction

Anxiety and depression are two of the most common psychiatric disorders worldwide, with deleterious effects on the quality of life and economic burden of patients and their families [1]. Pharmacological treatment of anxiety disorders includes diverse drugs, such as benzodiazepines and antidepressant drugs endowed with anxiolytic actions, such as selective serotonin reuptake inhibitors [2], and for depressive disorders, pharmacological treatment includes selective serotonin reuptake inhibitors, serotonin/noradrenaline reuptake inhibitors, tricyclic antidepressants, and some GABAergic neurosteroids [3]. Although there are several types of anxiolytic and antidepressant drugs, most of them have some clinical limitations. For example, anxiolytics such as benzodiazepines have restricted use due to common side effects, including somnolence, motor impairment, sedation, dependence, and pharmacological tolerance in the long term [4].

Moreover, antidepressant drugs require at least three to four weeks of treatment to produce their therapeutic effects, which is associated with the long-term establishment of neuroplasticity [5]. Additionally, the side effects of antidepressant drugs reduce treatment adherence. Consequently, patients will seek alternatives to improve their mental health. Therapeutic options have been found in traditional antique medicines from ancient cultures around the world, using medicinal plant extracts to produce anxiolytic and antidepressant effects, which are currently related to actions on diverse neurochemical, neuroanatomical, and neurotrophic processes [6,7,8,9]. Unfortunately, most lack controlled preclinical and clinical studies that validate their potential therapeutic effects and better control side effects that could impair patient health. There are important codices and documents of ancient cultures that contain different methods for the treatment of diverse diseases. Interestingly, some cultures such as Olmec, Maya, Zapotec, Teotihuacan, Mixtec, Toltec, and Mexica (or Aztec) described symptoms that, in the context of modern medicine, can be identified as anxiety and depression disorders, among other psychiatric disorders [10,11].

Pre-Columbian Mesoamerican cultures used plants with medicinal properties described in the *Libellus de Medicinalibus Indorum Herbis* codex and the Historia General de las Cosas de la Nueva España, from 1552 and 1557, respectively. These works describe the use of cihuapahtli or zoapatle, a plant from the genus *Montanoa*, authenticated as *Montanoa tomentosa*, along with two other species, *Montanoa frutescens* and *Montanoa grandiflora*, which are recommended for the treatment of some disorders in women associated with the reproductive state [12,13]. Furthermore, the aqueous crude extract of these plants was used to treat anxiety and depression symptoms [14]. Currently, preclinical and clinical studies have identified the beneficial effects of these plants on reproductive physiology and the control of anxiety and depression symptoms [15,16,17,18,19] but some toxic effects have also been identified [20,21]. All these biological effects have been associated with their principal phytochemicals, including triterpenes, diterpenes, monoterpenes, and sesquiterpenes, as well as sterols, flavonoids, and alkaloids.

The main objective of this review is to describe and discuss the potential anxiolytic and antidepressant-like effects of the three species of *Montanoa* genus plants mentioned above, as well as their ethnobotanical, pharmacological, toxicological, and phytochemical characteristics based on historical, descriptive, and experimental studies. This review aims to show the importance of traditional antique medicine in the scientific study of alternative therapies to treat psychiatric disorders and to identify potential side effects that could impair individual health.

## 2. Medicine in Pre-Columbian Mesoamerican Cultures

Human settlements established in the Valley of Mexico 15,000–12,000 years ago used primitive medicine. Then, later cultures such as the Olmec, Teotihuacan, Maya, Zapotec, Purépecha, Toltec, and Azteca made significant developments in different areas of science [22]. They developed an ancient medicine called ticiotl [23], based on materials from plants, animals, and minerals, as well as magic and religious treatments according to the cultural aspects of their cosmovision regarding health and disease [24,25]. Tenochtitlan was home to the Aztecs, one of the most important empires of Mesoamerica, which aroused great interest among Spanish colonizers. For this reason, most information on sociocultural and medical practices is mainly from the Aztecs [26,27].

**Figure 1:**
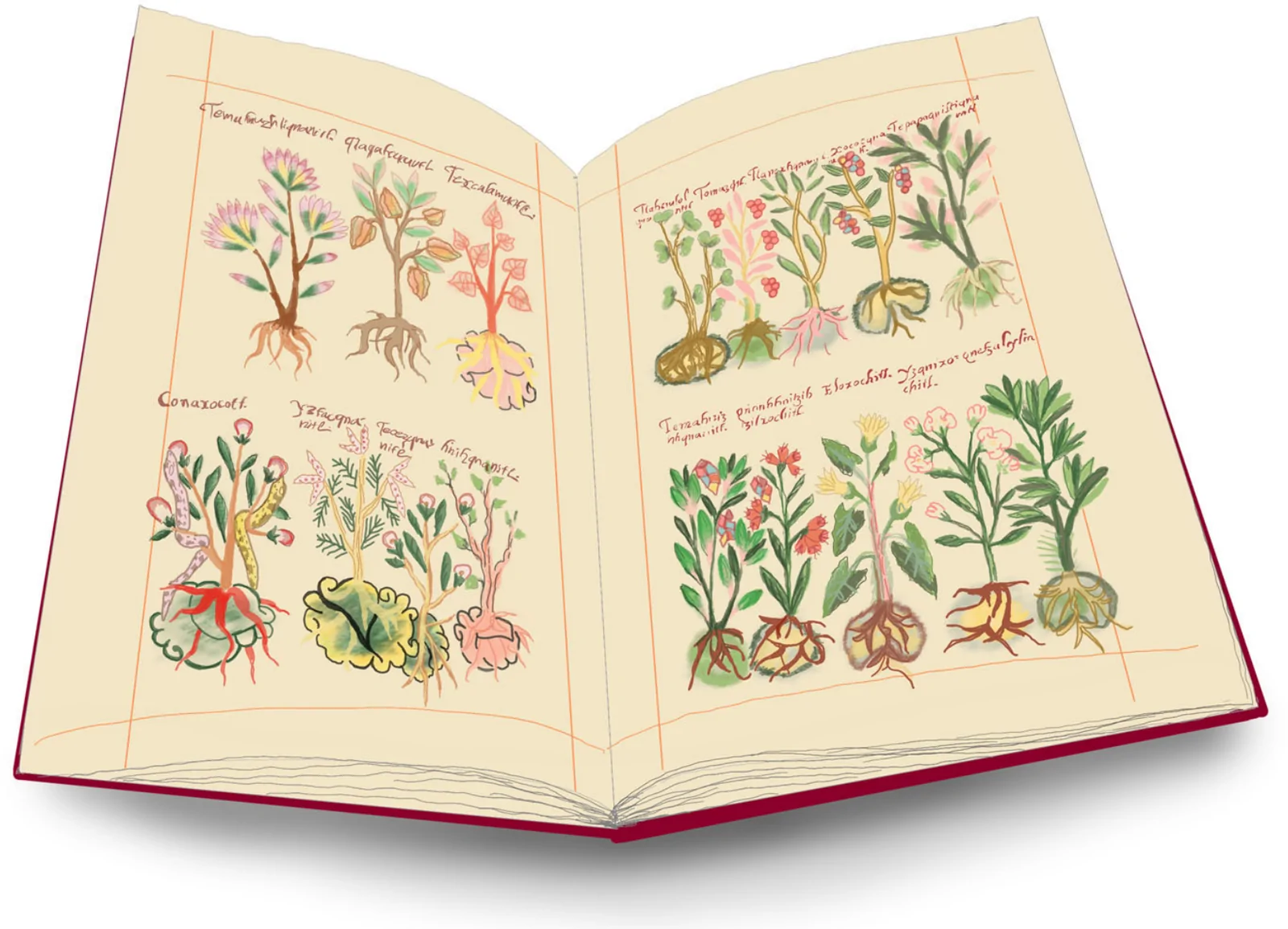
*Libellus de Medicinalibus Indorum Herbis*. This codex contains information about the extensive use of medicinal plants by pre-Columbian Mesoamerican cultures, including illustrations with natural pigments. Digital drawing by Jonathan Cueto-Escobedo, based on folios 38v and 39r of the original digitized Badian Codex [28].

Pre-Columbian Mesoamerican medicine used by Aztec physicians (titici) was compiled in ancient documents written by Franciscan monks who arrived in New Spain during the Spanish colonization. Martin de la Cruz, an indigenous physician, described its curative methods in Nahuatl, and Juan Badiano wrote them in Latin in the *Libellus de Medicinalibus Indorum Herbis* (1552), currently known as the de la Cruz-Badian Codex (Figure 1). The importance of this codex primarily lies in its deep knowledge of medicinal plants and its artistic content, as medicinal plants were illustrated with their common names in Nahuatl, along with descriptions of their effects and therapeutics [25].

Francisco Hernandez (“protomedico” in the medical court of Felipe II of Spain), Fray Alonso de Molina, and Fray Bernardino de Sahagun collected information about the medicine of pre-Hispanic Mexico. In 1557, Bernardino de Sahagun collected information about the native people of pre-Hispanic cultures and wrote the Florentine Codex, or Historia General de las Cosas de la Nueva España, in Nahuatl [29]. In 1570, Francisco Hernández studied indigenous Mesoamerican medicine and wrote the “Historia de las Plantas”, highlighting the wide knowledge of the use of plants for treating and preventing diverse diseases, compiling knowledge of pre-Columbian Mesoamerican cultures on medicinal plants.

### The Concepts of Health and Disease in Pre-Columbian Mesoamerican Medicine

The concept of health in pre-Columbian Mesoamerican cultures refers to an equilibrium between the individual, the environment, and the gods, while disease appears when disequilibrium occurs [24]. The destiny birthmark, present at birth, was also considered to influence health and disease. Pre-Hispanic priests (Tonalpohuque) consulted the book of fate of humans (Tonalamatl) to know the life expectancy, health, and diseases of people in the present and future [27]. The titici considered disease as part of the hot–cold polarity of their dual cosmovision [27,30], which resembles, to some degree, the Hippocratic–Galenic theory of the humors.

Antique Mesoamericans observed symptoms of mood disorders, which modern medicine would identify as anxiety and depression [11]. Experts have identified facial characteristics in pre-Columbian ceramic figures that suggest representations of depressive states [31], and several codices describe emotional states that could correspond to depressive disorders. For example, the Historia General de las Cosas de la Nueva España mentions that pre-Columbian titici identified two types of “depression”, the agitated (Tlahuilolocoyotl) and the slowed (Xolopiyotl), which were considered a disease of the heart (yollotl). Similarly, a low emotional state is described in the Tratado Breve de Medicina [31]: “… it is a common disease in many (people), afflicts and tortures them with serious accidents, when the men and women have twenty years old, they complain of melancholia and of the heart…”.

Pre-Columbian Mesoamerican medicine has also described some therapeutic methods based on medicinal plants. The *Libellus de Medicinalibus Indorum Herbis* describes the treatment for “depression”, referred to as a remedy for the “black blood” (*nigri remedium sanguinis*); it is prepared with aromatic flowers and patients are recommended to “…walk in shady places, drink pulque (an alcoholic beverage from maguey), practice joyful activities, sing, and play a musical instrument…”.

**Figure 2:**
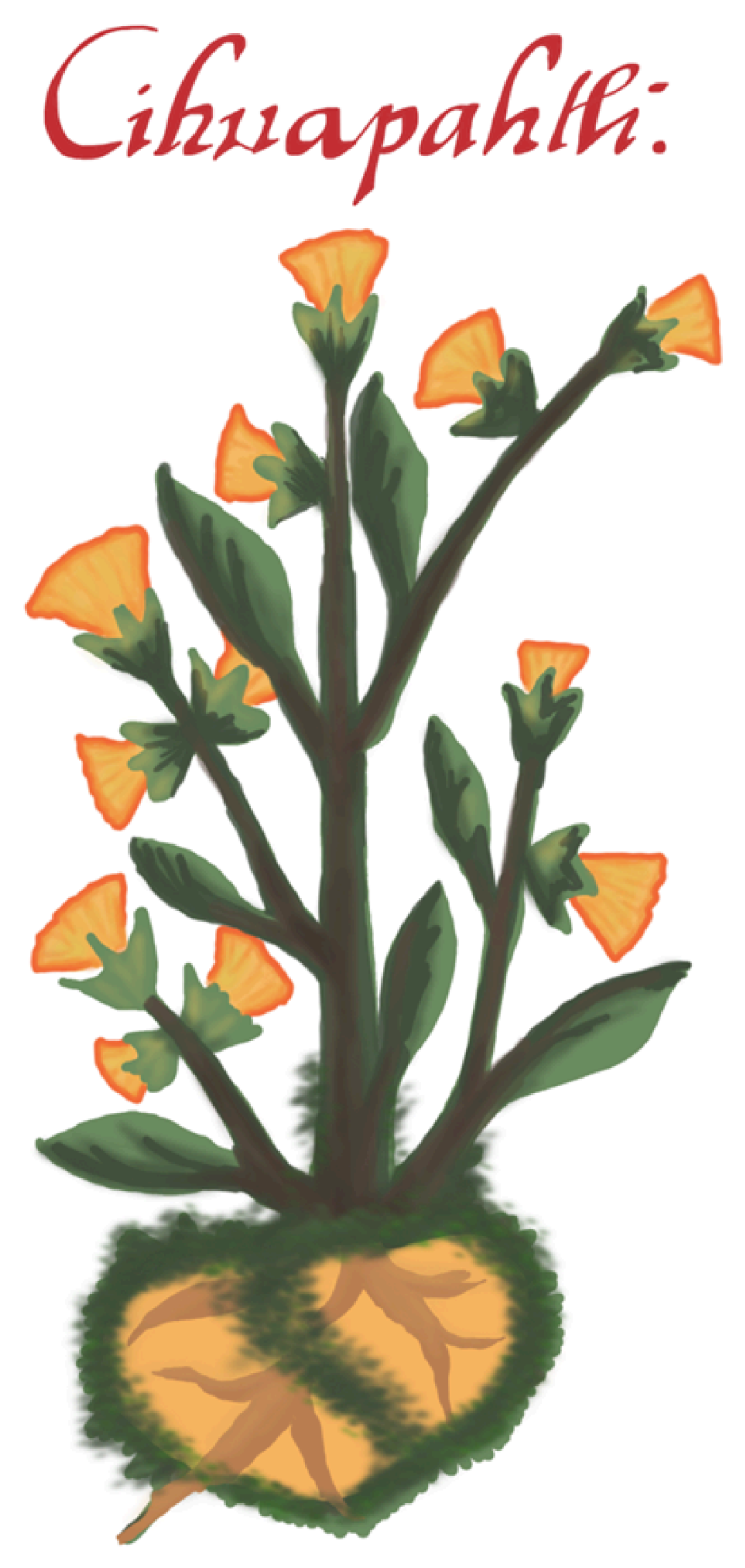
Zoapatle or Cihuapahtli from the *Libellus de Medicinalibus Indorum Herbis*. Cihuapahtli (cihua = woman, and pahtli = medicine) in Nahuatl means “woman’s medicine”. Authenticated as *Montanoa tomentosa*, the aqueous crude extract of which was used to treat reproductive impairments and mood disorders. Digital drawing by Jonathan Cueto-Escobedo, based on the original Badian Codex.

The *Libellus de Medicinalibus Indorum Herbis* describes some medicinal plants for the treatment of mood alterations and some reproductive ailments such as Zoapatle or Cihuapahtli in Nahuatl (Figure 2); a group of plants of the Asteraceae family—the *Montanoa tomentosa*, *Montanoa grandiflora*, and *Montanoa frutescens*—are used in aqueous crude extract mainly as a contraceptive agent during early stages of pregnancy [12,13,14,32] and to induce rhythmic uterine contractions at birth. Mood and “nervous” disorders (anxiety-like behavior in the context of modern medicine) are also treated with the aqueous crude extract of cihuapahtli, and early reports by Ximenez [14] mention that “… cihuapahtli resolves the mood changes and nerves in an admirable form…”.

Relatively recent studies on the pharmacological effects of Cihuapahtli have identified its potential therapeutic use in reproductive disorders in clinical research, as well as its anxiolytic- and antidepressant-like effects in preclinical studies. The partial mechanisms of action of these plants have been studied, and the three Montanoa species (i.e., *M. tomentosa*, *M. grandiflora*, and *M. frutescens*) share pharmacological effects that activate some neurotransmission systems, such as the GABAergic system through the activation of the GABA_A_ receptor. Additionally, the activation of the oxytocinergic system in the paraventricular nucleus has been identified. The activation of these neurocircuits suggests that components of these plants could be used to develop treatments for anxiety and depression symptoms.

## 3. Ecological and Ethnobotanical Generalities of *Montanoa* Genus Plants in America

Cihuapahtli belongs to the Asteraceae family, which comprises more than 1535 genera and 23,000 species of composite plants [33], containing more than 25% of all existing angiosperm species [34], including numerous species of economic [35], ornamental, and pharmaceutical importance [36].

The *Montanoa* genus encompasses approximately 33 species, 4 subspecies, and 6 varieties (Table 1) [37]. Its distribution is geographically wide, from the central zone of Mexico to the central zone of Colombia [38]. Two phylogenetic lineages contain approximately half of the species. One clade contains mainly species from central Mexico (Mexican clade), while the other contains Mesoamerican and South American species (Mesoamerican/South American clade) [39]. Within the Mexican endemic clade, the species *M. frutescens*, *M. grandiflora*, and *M. tomentosa* are valued for their medicinal effects [15,18,40,41].

The species of the *Montanoa* genus are widely distributed in Mexico. According to the records of the New York Botanical Garden Steere Herbarium NY, 1954, *M. frutescens* is distributed throughout the country from the state of Sinaloa in the north [42], as well as in the central states of Guanajuato, Michoacán, the State of Mexico, Morelos, Mexico City, and Puebla. In the south, it is distributed in the states of Guerrero, Oaxaca, Chiapas, and Veracruz. According to the collection sheets, the altitude distribution ranges from 2800 (Guerrero state) to 1050 m above sea level (Mexico City). *M. frutescens* is found in various habitats, such as pine–oak forest, cloudy mountain forest, tropical deciduous forest, and secondary scrub. The species *M. grandiflora* is distributed throughout Colima, Jalisco, Michoacán, Mexico City, Morelos, Puebla, Guerrero, Chiapas, and Veracruz states. This species is found between 160 m above sea level in the municipality of Ocosingo, Chiapas, and up to 2630 m above sea level in the Altamirano hill of the Monarch Butterfly Biosphere Reserve. It has been found in diverse types of vegetation, such as mountain cloud forest, deciduous forest, high subcaducifolious forest, and cool pine–oak forest on steep mountain slopes, as well as in roadside ditches and fencerows in cultivated grazed hills.

*M. tomentosa* has two subspecies, *rosei* and *tomentosa*, and four varieties: *microcephala*, *tomentosa*, *xanthifolia*, and *xanthiifolia*. These varieties are distributed in Sonora, Sinaloa, Jalisco, Michoacán, Querétaro, San Luis Potosí, Morelos, Puebla, Guerrero, Oaxaca, Chiapas, and Veracruz states, as well as in Mexico City [37]. *M. tomentosa* is particularly important because it was referred to in ancient Mexican medicine as a remedy for facilitating parturition and ameliorating puerperal symptoms, serving as a contraceptive agent and a remedy for mood disorders. A significant number of studies have identified the chemical compounds contained in *M. tomentosa*, *M. frutescens*, and *M. grandiflora* that produce important biological and pharmacological activities.

## 4. Phytochemical Constituents of *M. tomentosa*, *M. grandiflora*, and *M. frutescens*

**Figure 3:**
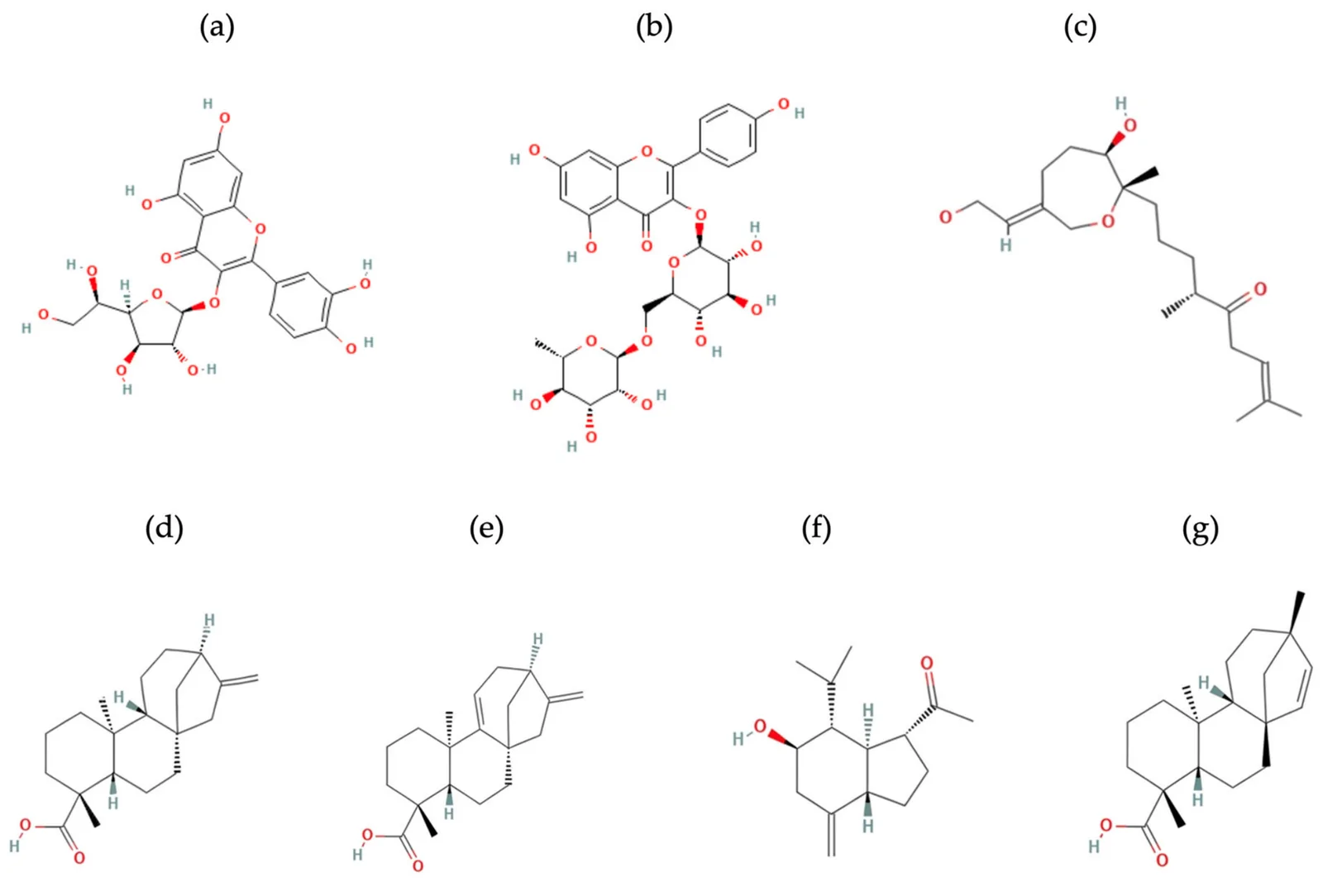
(**a**) Isoquercitrin: 3-[(2S,3R,4R,5R)-5-[(1R)-1,2-dihydroxyethyl]-3,4-dihydroxyoxolan-2-yl] oxy-2-(3,4-dihydroxyphenyl)-5,7-dihydroxychromen-4-one. Its ability to mitigate the symptoms of anxiety or depression has been documented. (**b**) Nicotiflorin: 7-dihydroxy-2-(4-hydroxyphenyl)-3-[(2R,3S,4R,5R,6S)-3,4,5-trihydroxy-6-[[(2R,3R,4R,5R,6S)-3,4,5-trihydroxy-6-methyloxan-2-yl] oxymethyl] oxan-2-yl] oxychromen-4-one. It primarily acts as a powerful antioxidant, anti-inflammatory, and neuroprotective agent. (**c**) Zoapatanol: (6R)-9-[(2S,3R,6E)-3-hydroxy-6-(2-hydroxyethylidene)-2-methyloxepan-2-y1]-2,6-dimethylnon-2-en-5-one. It is a sesquiterpenoid, an isomer of montanol found in the *Montanoa* genus plant. It has a powerful uterotonic effect; that is, it stimulates the contraction of the uterine muscles and may be associated with oxytocinergic activation in the hypothalamus. (**d**) Kaurenoic acid: (1S,4S,5R,9S,10R,13R)-5,9-dimethyl-14-methylidenetetracyclo [11.2.1.01,10.04,9] hexadecane-5-carboxylic acid. It has been studied for its medicinal properties and seems to have anti-inflammatory, antiulcerogenic, antitumor, antinociceptive, antimelanoma, anti-lipoperoxidation, antioxidant, and antimicrobial properties. It may be associated with oxytocinergic activation in the brain. (**e**) Grandiflorenic acid: (1S,4S,5R,9R,13R)-5,9-dimethyl-14-methylidenetetracyclo [11.2.1.01,10.04,9] hexadec-10-ene-5-carboxylic acid. It is a tetracyclic diterpenoid with the formula C_20_H_28_O_2_, which exhibits anti-inflammatory, antibacterial, antileishmanial, and wound-healing properties. It has a role as an antibacterial agent, an anti-inflammatory agent, an antileishmanial agent, a vulnerary, and a plant metabolite. It is a monocarboxylic acid, an ent-kaurane diterpenoid, and a tetracyclic diterpenoid. It may be associated with oxytocinergic activation in the brain. (**f**) Sesquiterpene: 1-(6-Hydroxy-7-(propan-2-yl)-4-methylidene-2,3,3a,4,5,6,7,7a-octahydro-1H-inden-1-yl) ethanone. (**g**) Monoginoic acids: (1R,4S,5R,9S,10S,13S)-5,9,13-trimethyltetracyclo (11.2.1.01,10.04,9) hexadec-14-ene-5-carboxylic acid. They could be involved due to their anxiolytic effects. PubChem. National Library of Medicine: https://pubchem.ncbi.nlm.nih.gov.

*M. tomentosa* is described in the ancient Mesoamerican codices. This plant was used to relieve some difficulties of pregnant women during childbirth, treat amenorrhea, and induce abortion [29,43]. It was also used to treat mood disorders such as anxiety and depression. Recent preclinical studies have identified aphrodisiac properties of this plant and other species, such as *M. frutescens* and *M. grandiflora* [16,44,45]. This last effect was not described in ancient Mesoamerican medicine, but it could be related to regulating the male emotional state and improving sexual behavior. Considering these potential therapeutic effects, diverse studies have attempted to identify and isolate the bioactive compounds contained in these three plant species. In this regard, since the early 1970s, the isolation and structural elucidation of the secondary metabolites contained in different extracts of *M. tomentosa*, *M. frutescens*, and *M. grandiflora* [46,47,48], as well as other *Montanoa* species not described in the pre-Columbian Mesoamerican codices, have been investigated. The principal phytochemicals identified in those extracts have been diterpenes, monoterpenes, sesquiterpenes, and triterpenes [49], as well as flavonoids and alkaloids [19]. Preclinical studies of these secondary metabolites have shown that they are responsible for the pharmacological actions related to their therapeutic effects, which are shared by the three *Montanoa* species. The principal active constituents of the three *Montanoa* species discussed in this review are illustrated in Figure 3.

### 4.1. Montanoa tomentosa Extracts

Different extracts of this plant have been phytochemically characterized to elucidate the chemical compounds responsible for its contraceptive and antifertility activity. The infusion prepared with leaves of *M. tomentosa* contains sesquiterpenoid oxepane [50]; 21-normontanol [51]; and zoapatanolides A, C, and D [52]. In addition, the presence of monoginoic acid and kaurenoic acid in the root extract of this plant was identified [53]. Likewise, some flavonoids were identified in both extracts, including nicotiflorin and isoquercitrin [54], as well as oxepane 3-C-methyl-α-D-allofuranose, which is a 2-zoapatanol analog [55].

Monoterpenes such as sabinene, α-pinene, α-thujene, and α-gurjunene were detected in the infusion prepared with fresh leaves and flowers of *M. tomentosa*, as well as some sesquiterpenes such as caryophyllene and germacrene D [56,57]. The crude extract of leaves and roots of this plant contains diterpenes such as kaurenoic acid, grandiflorenic acid, and monoginoic acid [58].

When the leaves were percolated and the product analyzed via proton nuclear magnetic resonance, some sesquiterpene lactones were identified, such as zoapatanolides A and B [59] and guaiamolide compounds such as zoapatanolides C and D [60]. Additionally, terpenoids such as tomentanol [61], kaurene diterpenes such as kaur-16-en-19-oic acid, kaur-9(11)16-dien-19-oic acid, angeloylgrandifloric acid, monoginoic acid, and grandifloric acid [62] were identified. The oil volatiles borneol acetate, β-cubebene, and β-caryophyllene were present in the crude extract prepared with milled *M. tomentosa* [63]. In *M. tomentosa* extract, sesquiterpenes lactones such as 4β,5α-Epoxy-8α-isobutyryloxy-trans-germacr-1(10)-en12,6β-olide, 4β,5α-Epoxy-7α-hydroxy-8α-isobutyryloxy-trans-germacr-1(10)-en12,6β-olide, (1R,4S)-1,5-Cyclo-5α-hydroxy-8α-isobutyryloxy-7α,10α-oxagermacran-12,6β-olide, and 4β-Hydroxy-8α-isobutyryloxy-7α,10α-oxyguaian-12,6β-olide have also been detected [64].

Moreover, the methylene chloride extract from the leaves of *M. tomentosa* contains zoapatanol sesquiterpenoids such as pretomentol, pretomentol acetate, prezoapatanol, and pretomexanthol [65]. In addition, the infusion prepared with the leaf, stem, and inflorescence of this plant reported the presence of sesquiterpenes lactones, including 3α-epoxypumilin and 8-acetyl-9-desacylpumilin-9-methacrylate, 8α-methacryloyloxy-4β,5α-epoxy-trans, germacra-1(I0)en-12,6β-olide, 8α-(2,3-Epoxy-2-methylbutyryloyloxy)4β,5α-epoxy-trans, germacra-1(I0)en-12,6β-olide, 7α-Hydroxy-8-methacryloyloxy-4,5-epoxy-trans, germacra-1(I0)en-12,6-olide, 7α-Hydroxy-8-(2,3-epoxy-2-methylbutyryloyloxy)-4,5α-epoxy-trans, germacra-1(I0)en 12,6-olide, 7α-Hydroxy-8-methacryloyloxy-4,5-epoxygermacra-10(I4)en-12,6-olide, and 7α-Hydroxy-8-methacryloyloxy-4,5,1,10-diepoxygermacra-12,6-olide [66,67], as well as some terpenoids such as tomexanthin and Zoapatanol [68]. The hexanoic extract of the leaves contains kaouradienoic acid [69], while the petroleum extract contains sesquiterpene lactones such as zoapatanolides A, C, and D, and pumilin, in addition to diterpenes zoapatols A and B, guaianolide, zoapatanolide F, zoapatanol, and montanol [70,71].

Finally, the petroleum ether extract prepared with the areal parts of *M. tomentosa* showed the presence of terpenoids such as 8α-(4-acetoxymethacryloyloxy)-3α,9β-dihydroxy-1(10)E,4Z,11(13)-germacratrien12,6α-olide, 8α-(2′E)-(2′-acetoxymethyl-2′-butenoyloxy)-3α,9β-dihydroxy-1(10)E,4Z,11(13)-germacratrien-12,6α-olide, and zoapatanolide A [72]. Other chemical compounds identified in alcoholic, organic solvent, and aqueous extracts confirm the presence of zoapatanol, montanol, nicotiflorin, isoquercitrin, tomexanthin, tomentol, zoapatanolide A, kauradienoic acid, grandiflorenic acid, and methyl grandiflorenate. Recently, the glandular trichomes of this plant’s leaves were analyzed, which revealed the presence of kaurenoic acid, grandiflorenic acid, β-eudesmol, and valencene [57].

### 4.2. Montanoa frutescens Extracts

In contrast to *M. tomentosa*, the phytochemical profile of *M. frutescens* extracts has been scarcely studied. However, preliminary phytochemical analysis of the aqueous crude extract of this plant has shown the presence of flavonoids, alkaloids, sesquiterpenoids, and terpenes [19]. A petrol extract prepared with fresh leaf, flower, and stem materials contained caryofillene, taraxasterol acetate, kaurenic acid, and its corresponding 15α-isovaleriate [73]. Montafrusin and montafrusin acetate have been obtained from guaiacol methane extract prepared with the leaves, flowers, and stems of *M. frutescens* [74]. Additionally, other sesquiterpenoids, such as montafrusin and montafrusins A and B, some diterpenes, such as kaurenoic acid, and eudesmanolides, such as montafrusins C, D, E, and F, have been identified in different extracts prepared from the aerial parts of *M. frutescens* [52,73,74].

Hexane extract from leaves of this plant has shown the presence of kaurenoic acid and its 15α-isovalerate; subsequently, the presence of 15α-hydroxy-dihydro kaurenoic acid has been identified, as well as the methyl ester of 15-keto-dihydro-kaurenoic acid [75].

### 4.3. Montanoa grandiflora Extracts

A preliminary phytochemical study of the aqueous crude extract prepared from the aerial parts of this plant showed the presence of flavonoids, alkaloids, sesquiterpenoids, terpenes, and sterols [19]. Specifically, a chloroform extract prepared with fresh leaves of *M. grandiflora* showed the presence of the sesquiterpene lactone 6β-hydroxy-germacradien-8,12-olide [60]. In addition, a nonpolar extract prepared with leaves of *M. grandiflora* showed the presence of the terpene caryophyllene oxide and triterpene lup-20(29)-en-3-ol acetate [76].

Analysis of the essential oil composition from the inflorescence has shown the presence of terpene compounds such as α-pinene, limonene, eucalyptol, terpineol, geranyl acetate, and terpenyl acetate. Similarly, the essential oil of the stem of this plant contains α- and β-pinene, limonene, eucalyptol, menthol, citronellal, and thymol. In contrast, the essential oil of the leaves contains α-pinene, α-phellandrene, champhene, limonene, eucalyptol, citronellal, terpineol, guaiacol, methyl eugenol, and manol [77].

In summary, the phytochemical profile of the tree species *Montanoa* shows the presence of flavonoids, alkaloids, sesquiterpenoids, and terpenes, which probably contribute to the medicinal properties reported for these plants. However, some terpenes could also be involved in their toxic effects, particularly diterpene kaurenoic acid and associated compounds. Therefore, specific studies are necessary to identify or discard the pharmacological effects of *Montanoa* extracts and their secondary metabolites for the development of alternative therapeutics for the treatment of psychiatric disorders such as anxiety and depression.

## 5. Anxiolytic-like Effects of Some *Montanoa* Genus Plants

Anxiety disorders are increasing exponentially due to different factors that include biological, psychological, and social stressors [78,79], which affect approximately 10% of the world’s population [80]. These disorders negatively impact patients’ quality of life, as well as their labor and productivity [81]. Phobias are among the most common anxiety disorders worldwide, with a prevalence of 10.3%, followed by panic disorders at 6% and generalized anxiety disorders at 2.2% [82]. However, the COVID-19 pandemic has increased the prevalence of anxiety disorders [83]. These disorders are characterized by psychological, physical, and emotional symptoms, including excessive fatigue, irritability, concentration impairment, sleep disorders, excessive sweating and worry, muscle tension, and increased vigilance that may be of short or long duration [84], which could depend on the lifestyle of each patient [85].

Anxiety disorders involve neurochemical alterations in the amygdala, hippocampus, and prefrontal cortex, among other brain structures. The neurochemical mechanism involved in anxiety disorders includes reduced GABAergic neurotransmission, which affects the proper function of the GABA_A_/benzodiazepine receptor complex [86] and is associated with alterations in neuronal connectivity in patients [84]. Additionally, anxiety disorders also involve multiple alterations in other neurotransmission systems, such as serotonergic and noradrenergic systems, alterations in the concentration of neurosteroids and the production of neurotrophic factors, in addition to some neuroinflammatory and oxidative processes [87,88,89,90,91]. In this manner, the pharmacotherapy of anxiety disorders is destined to re-establish the neurochemical function of the brain to reduce anxiety symptoms [92,93]. The principal anxiolytic drugs include benzodiazepines such as diazepam. However, the use of this kind of drug may produce diverse side effects that include sedation, muscle relaxation, slower psychomotor skills, dependence, and pharmacological tolerance in the long term [94]. Nonetheless, recent studies have highlighted the increase in the use of benzodiazepines, leading to a parallel rise in the rate of misuse and overdose [95]. For this reason, the use of some antidepressant drugs, such as selective serotonin/noradrenaline reuptake inhibitors, has increased for the treatment of anxiety disorders, considering their significant therapeutic efficacy and reduced side effects [96].

Additionally, hormone replacement therapy based on estradiol and progestins has been used in anxiety associated with hormonal changes during the reproductive cycle of women. However, this type of hormonal therapy, particularly with estrogens, is limited in women with a family history of estrogen-dependent cancer, in addition to its potential side effects on the cardiovascular system [97]. Thus, some patients with anxiety disorders associated with hormonal changes are administered, as an alternative therapy, the use of phytoestrogens isolated from soybean (*Glycine max* L.) and some nutritional supplements that include polyphenols and phytoestrogens from different plants [19]. Some patients even use plant extracts based on the traditional medicine of different communities around the world as alternative therapies.

As previously mentioned, *M. tomentosa*, *M. frutescens*, and *M. grandiflora* have been used to treat some disorders associated with the reproductive stage in women [98,99], and these have been evaluated in preclinical and clinical research. Nonetheless, studies on the other properties, such as their anti-anxiety and antidepressant effects, attributed to *Montanoa* genus plants have been scarce, but the results have been promising.

In 2004, Carro-Juárez and collaborators began studying the effects of *M. tomentosa* extracts on sexual behavior in male rats, showing that they improve sexual behavior and produce aphrodisiac effects [44], which could be partially associated with its relaxing and anxiolytic-like effects. In this study, the authors evaluated different doses (38, 75, and 150 mg/kg) of the aqueous crude extract of the aerial parts of *M. tomentosa* on copulatory, noncopulatory, and genital anesthesia in male rats and its effects on sexual behavior. The extract facilitated the expression of sexual and arousal behavior in the three groups of rats, which suggested an aphrodisiac effect. In support of this hypothesis, in sexually experienced spinal male rats, an intravenous administration of 25 mg/rat of the aqueous crude extract of *M. tomentosa* activated an ejaculatory rhythmic motor pattern, penile erection, penile movements with the expulsion of urethral contents, and, in some cases, the expulsion of seminal plugs, similar to those induced via urethral stimulation and 0.5 IU/kg oxytocin injections [45]. These effects were blocked by the previous administration of hexamethonium, a selective oxytocin antagonist, suggesting that *M. tomentosa* extract in male rats possesses aphrodisiac effects, acting as an oxytocic agent. Interestingly, the aqueous crude extracts of *M. frutescens* and *M. grandiflora* also produced similar effects on sexual behavior and aphrodisiac effects such as those produced by *M. tomentosa* [16]. This shows that chemical compounds contained in *Montanoa* plant extracts may cross the blood–brain barrier and exert pharmacological action on the central nervous system, modulating some aspects of sexual behavior by acting on the oxytocinergic system. This is important because improved sexual behavior may be produced by anxiolytic drugs [100], and oxytocin has been reported to produce anxiolytic-like effects in preclinical [101] and clinical research [102,103]. This could help explain the anxiolytic effects reported for *Montanoa* genus plants in pre-Columbian Mesoamerican medicine.

Indeed, preclinical research has been conducted on the anxiolytic effects and the mechanism of action of some extracts prepared with the aerial parts of those plants, finding that Montanoa extracts could have potential application in the development of phytomedicines for the treatment of anxiety disorders.

The effect of three doses of the aqueous crude extract of the aerial parts of *M. frutescens* and *M. tomentosa* (25, 50, and 75 mg/kg) was evaluated in adult male Wistar rats on anxiety-like behavior in the elevated plus maze and open field test and compared with diazepam. The results showed that 25 and 50 mg/kg of *M. frutescens* and 50 mg/kg of *M. grandiflora* extract produced anxiolytic-like effects similar to those of 1 and 2 mg/kg diazepam. In comparation, 75 mg/kg of both plant extracts lacked anxiolytic-like effects, producing hypoactivity in the open field test, as did 4 mg/kg diazepam [15]. Interestingly, previous administration of picrotoxin, a noncompetitive antagonist in the chloride ion channel of the GABA_A_ receptor, blocked the anxiolytic-like effect produced by 50 mg/kg of *M. frutescens* and *M. grandiflora* extracts [15]. The abovementioned results suggest that the GABAergic system is involved in the anxiolytic-like effects of the compounds contained in *Montanoa* plants. Thus, steroid hormones such as estradiol and progesterone in females regulate anxiety-like behavior, particularly during the ovarian cycle, by acting on the GABA_A_ receptor. In the proestrus–estrus phase of the ovarian cycle, when the concentration of ovarian hormones increases, anxiety-like behavior decreases.

In contrast, during metestrus–diestrus, when the concentration of ovarian hormones is low, anxiety-like behavior increases [104]. Interestingly, the administration of 25 and 50 mg/kg *M. frutescens* and 50 mg/kg *M. grandiflora* extracts reduces the naturally occurring anxiety-like behavior during the metestrus–diestrus phase, similar to the administration of diazepam in the elevated plus maze, without disrupting spontaneous motor activity [18]. These results show that both Montanoa plant extracts possess anxiolytic-like effects that depend on the ovarian cycle phase and support the therapeutic properties described in ancient medicine to reduce “anxious states” in females.

Similarly to *M. frutescens* and *M. grandiflora*, *M. tomentosa* extracts also produce anxiolytic-like effects. Different doses of lyophilized *M. tomentosa* extract (1.5, 3, 6, and 12 mg/kg) were evaluated for anxiety-like behavior based on burying behavior, elevated plus maze, and hole-board tests in adult male Wistar rats. The results showed that 3 mg/kg of lyophilized extract produced anxiolytic-like effects in the three behavioral tests without producing significant changes in general locomotor activity. In comparison, higher doses (12 mg/kg) produced sedative effects, increasing motor incoordination in the rotarod [17]. These effects were similar to those produced by muscimol, a potent agonist of GABA_A_ receptors. Additionally, the mechanism of action involved in the anxiolytic-like effects of the lyophilized extract of *M. tomentosa* has been determined to involve the GABA_A_ receptor, since the administration of picrotoxin (a blocker of the chloride ion channel), bicuculline (a selective competitive antagonist for the binding site of the γ-aminobutyric acid), and flumazenil (a selective competitive antagonist for the benzodiazepine binding site) canceled the anxiolytic-like effect of *M. tomentosa* extract [17]. The aforementioned results indicate that anxiolytic-like effects produced by this plant are established through action on the GABA_A_ receptor.

It is important to highlight that the anxiolytic-like effects of *M. tomentosa* extracts in female rats depend on the hormonal stage. For example, in the experimental model of surgical menopause in female rats induced via long-term ovariectomy, there is an increase in anxiety-like behavior [105,106] associated with a reduced concentration of ovarian hormones. In this model, the administration of 12.5, 25, and 50 mg/kg of the aqueous crude extract of *M. tomentosa* showed that only 50 mg/kg of extract significantly reduced anxiety-like behavior similar to the administration of diazepam in the elevated plus maze, without affecting spontaneous locomotor activity in the open field test [40].

Additionally, it was reported that the anxiolytic-like effects of *M. tomentosa* extracts depend on the endocrine state of female rats. Different doses (0.38, 0.75, 1.5, and 3 mg/kg) of *M. tomentosa* lyophilized extract were evaluated in female rats during the proestrus/estrus and diestrus phases and in three-week post-ovariectomized rats, compared with diazepam use. Only the 1.5 mg/kg dose produced anxiolytic-like effects in the diestrus phase and in ovariectomized rats, similar to diazepam; that is, the anxiolytic-like effect was identified only when low concentrations of ovarian hormones were detected. The anxiolytic-like effects produced by 1.5 mg/kg *M. tomentosa* extract in surgically menopausal rats were prevented by the antagonist picrotoxin, confirming GABA_A_ receptor participation in these actions [107]. Moreover, in an experimental model of premenstrual syndrome induced by progesterone withdrawal challenge in female rats, the administration of 0.38 and 0.75 mg/kg of *M. tomentosa* extract produced anxiolytic-like effects compared with the control group. In contrast, different doses of diazepam (0.12, 0.25, 0.5, and 1 mg/kg) were devoid of anxiolytic-like effects [107]. This result may be of special interest because *M. tomentosa* extract could be an alternative to the use of diazepam in ameliorating anxiety symptoms associated with premenstrual syndrome, contributing to the development of phytomedicines in this group of patients.

Altogether, these results show that the anxiolytic-like effect of the three species of *Montanoa* genus in male and female rats at different endocrine stages involves the GABAergic system, particularly the GABA_A_ receptor, since antagonists of this receptor block the anxiolytic-like effects of Montanoa extracts. Phytochemical analysis of the aqueous crude extracts of *M. frutescens*, *M. grandiflora*, and *M. tomentosa* conducted in previous studies has shown the presence of flavonoids, alkaloids, and sesquiterpenoids [19], in addition to the flavonoid rutin in *M. tomentosa* extract [107]. This is important because flavonoids target GABA_A_ receptors and produce several neurochemical and neuroanatomical changes in specific brain structures involved in the control of anxiety, such as the hippocampus, prefrontal cortex, amygdala, and raphe nucleus [108,109,110,111], which contribute to the anxiolytic- and antidepressant-like effects of these natural compounds [112]. In this sense, flavonoids could be considered a potential natural source for the development of phytomedicines for the treatment of anxiety disorders and other neuropsychiatric disorders.

## 6. Antidepressant-like Effects of Some *Montanoa* Genus Plants

Depression is a mood disorder that affects approximately 300 million people around the world and produces high disability [113]; it is characterized by a constant feeling of sadness, a lack of interest in pleasurable activities [114], sleep disturbances, feelings of loneliness, a lack of appetite, fatigue, cognitive impairment, and suicidal ideation [115,116,117], among other symptoms.

The etiology of depressive disorders is multifactorial and involves biological, genetic, psychological, and socioenvironmental factors that alter the concentration of neurotransmitters such as serotonin, noradrenaline, dopamine, and γ-aminobutyric acid (GABA), affecting the function of their receptors in brain structures related to emotional and cognitive processing [114,118,119]. In addition, neurotrophic factors, neuronal plasticity, oxidative stress, and neuroinflammation are also involved in the neurobiology of depression [120,121]. Recently, a close relationship between the gut microbiota and depressive disorders has been reported, since disruption of the microbiota predisposes individuals to neuroinflammatory processes that affect neuronal and neurochemical pathways involved in depressive symptoms [122,123]. The etiology of depressive disorders includes heritable genetic factors that predispose individuals to vulnerability to stressors, such as negative experiences during development, particularly during childhood and adolescence, which induce a deficient response to stress [124].

The pharmacotherapy of depression includes drugs that restore the neurochemical function of the brain, including selective serotonin reuptake inhibitors, selective noradrenalin/dopamine reuptake inhibitors, and tricyclic antidepressants, which, in the long term, induce neural plasticity and restore the function of neurotransmitter receptors [125]. Furthermore, in the long term, antidepressant drugs activate neurotrophic factors that impact the function of specific brain structures, such as the hippocampus and prefrontal cortex, reducing depression symptoms [126]. Recently, the neurosteroid allopregnanolone was introduced for the treatment of postpartum depression and post-traumatic stress disorders; this neurosteroid targets GABA_A_ receptors to re-establish GABAergic function [127,128]. Although antidepressant drugs are effective in the treatment of depression, not all patients respond favorably to their pharmacological and therapeutic effects [129]. Consequently, depressed patients seek therapeutic alternatives, including the use of herbal products and phytomedicines elaborated with plant extracts [130]. However, patients also use plant extracts recommended in ancient and modern traditional medicine as an alternative therapy.

Pre-Columbian Mesoamerican medicine used Montanoa plants to treat depression symptoms [14]. Considering the phytochemical profile of Montanoa plants, which contain alkaloids, terpenes, and flavonoids, their extracts may exert antidepressant-like effects. Thus, the polyphenol content in several plants can induce antioxidant and anti-inflammatory effects related to the antidepressant effects of drugs. In addition, some flavonoids activate the GABAergic system by targeting GABA_A_ receptors, as well as serotonergic receptors such as 5-HT_1A_ and 5-HT_2A_, re-establishing brain neurochemistry and inducing neuroplasticity in specific brain structures [108,131].

Pre-Columbian Mesoamerican medicine upheld *M. tomentosa* as a particularly useful treatment for mood disorders [14]. Interestingly, recent studies on animals have evaluated the antidepressant-like effect and the mechanism of action of some extracts of *Montanoa* plants. Their results suggest that Montanoa extracts, in addition to their anxiolytic-like effects, also produce antidepressant-like effects, which could contribute to the development of phytomedicines for the treatment of depressive disorders in particular groups of patients.

Although the antidepressant-like effect of Montanoa extracts has been scarcely studied, the results suggest potential therapeutic effects on depression. Adult male Wistar rats were evaluated for depression-like behavior with the forced swim test, and the effects of 25 and 50 mg/kg of *M. frutescens* and *M. grandiflora* on the 0th, 1st, 7th, 14th, 21st, and 28th days of treatment were compared with those of antidepressant drugs fluoxetine and Remotive^®^ (a phytomedicine prepared with *Hypericum perforatum* standardized extract). The results showed that 50 mg/kg of *M. frutescens* and *M. grandiflora* reduced the total time of immobility in the forced swim test from day 1 of treatment; however, this effect disappeared 24 h after treatment withdrawal. Interestingly, both antidepressant drugs (fluoxetine and Remotive^®^) reduced immobility after days 14 and 21 of treatment, respectively [19], but this effect lasted until 48 h after treatment withdrawal. This result is interesting since Montanoa extracts exert antidepressant-like effects faster than fluoxetine and Remotive^®^. Nonetheless, the effects of Montanoa also disappear sooner after the end of treatment. The early effect produced by Montanoa extracts could be associated with the activation of ionotropic receptors such as GABA_A_, considering that the behavioral effects of both extracts are modulated through this receptor. In contrast, the long-term effects produced by fluoxetine and Remotive^®^ could involve neuroplasticity effects that require 2 to 3 weeks to establish their antidepressant effects [132,133], like in humans. Therefore, Montanoa extracts may be used to control symptoms of depression in the future, while antidepressant drugs establish their antidepressant effect in the long term; however, specific studies are needed to confirm or discard this possibility.

In support of this hypothesis, the mechanism of action involved in the fast antidepressant-like effects of *M. frutescens* and *M. grandiflora* extracts was explored in adult male Wistar rats. The administration of 50 mg/kg of both extracts for 28 consecutive days produced antidepressant-like effects based on the results of the forced swim test, reducing the total time of immobility and increasing the latency to the first immobility, similar to the effects produced with the antidepressant fluoxetine. Interestingly, the antagonism of the GABA_A_ receptor with picrotoxin blocked the antidepressant-like effects produced by Montanoa extracts but not those produced by fluoxetine [19]. This result indicates that the antidepressant-like effects of Montanoa extracts are mediated through ionotropic receptors, while fluoxetine, as another antidepressant drug, acts on metabotropic receptors that activate neuroplasticity [134].

As previously mentioned, *M. tomentosa* extract was recommended in ancient Mesoamerican medicine as a prominent contraceptive agent during the early stage of pregnancy [12,14,33] and to induce rhythmic uterine contractions at birth, suggesting that chemical compounds contained in *Montanoa* extracts exert similar actions to those produced by oxytocin. This finding was supported by studies in which electrophysiological effects produced by Montanoa extracts were blocked by a previous administration of a selective oxytocin antagonist [45]. This is important because oxytocin has been reported to modulate emotional states [135] and produce some antidepressant-like effects [136,137]. Thus, Montanoa extracts may activate the oxytocinergic system and contribute to their attributed antidepressant effects. In fact, the acute administration of 50 mg/kg of *M. tomentosa* extract increases the number of Fos/oxytocin cells in the paraventricular and supraoptic nuclei in Wistar rats, which has been associated with antidepressant-like effects through the forced swim test. It is important to mention that the antidepressant-like effect produced by *M. tomentosa* is similar to that produced by fluoxetine; however, the activation of oxytocinergic cells was specifically detected in Montanoa extract-treated rats but not in fluoxetine-treated rats [41]. Additionally, this same effect was detected in rats acutely treated with an infusion of *M. grandiflora* and *M. frutescens* [138]. At the same time, chronic treatment showed no effects on oxytocinergic cells, suggesting that other neurotransmission systems could participate in their antidepressant-like effects [139].

Despite the few studies that have evaluated the antidepressant-like effects of Montanoa extracts, the results suggest that they could be a natural alternative to ameliorate depression symptoms, particularly in patients who do not respond to conventional serotonergic, noradrenergic, or dopaminergic antidepressant drugs. It is suggested that *Montanoa* extracts can be used to modulate other neurotransmitter pathways, such as GABAergic and oxytocinergic systems. This opens a new line of research on the therapeutic potential of medicinal plants and their chemical constituents, with the aim to contribute to the pharmacotherapy of depressive disorders.

## 7. Toxic Effects of Some *Montanoa* Genus Plants

In addition to the beneficial effects produced by the extracts prepared with the aerial parts of the *Montanoa* plants described in traditional Mexican medicine, some dangerous and toxic effects have also been reported and must be taken into consideration.

The extracts prepared with the aerial parts of *M. tomentosa* are probably the most studied with the aim to identify their beneficial and undesirable effects. The infusion prepared with the aerial parts of *M. tomentosa* has been reported to produce abortive effects in human and nonhuman females due to its uterotonic activity [12,14,32]. In pregnant and nonpregnant guinea pig and cat females, intravenous injection of *M. tomentosa* extract (dose not reported) produced an increase in uterine contractions and, particularly in female cats, produced a significant decrease in blood pressure (20–60 mm Hg) immediately after administration [140]. Additionally, the acute intravenous injection of 200 µL/kg aqueous crude extract and 400 µg/kg hexanoic extract prepared with the leaves of *M. tomentosa* significantly increased the uterine contractions in female guinea pigs, which was reproduced with the injection of 200 µg/kg kauradienoic acid, one of the active compounds found in this plant [57]. It is important to highlight that the uterine contractions produced by kauradienoic acid were greater in intensity and amplitude than those produced by aqueous crude and hexanoic extracts prepared with *M. tomentosa*. This suggests that kauradienoic acid may be one of the main causes of uterotonic activity and the abortive effects attributed to *M. tomentosa* extracts in Mesoamerican medicine.

In support of this hypothesis, 12 g/kg of aqueous extract prepared with leaves of *M. tomentosa* produced an abortive effect in female rhesus monkeys treated for four weeks [20]. However, 4, 8, and 12 mg/kg of the same extract administered for four weeks in pregnant female rats and 5 g/kg administered for two weeks to pregnant female dogs did not modify uterine contractions compared with nonpregnant rats and dogs, respectively [20]. This suggests that the effects on uterine contraction associated with *M. tomentosa* extracts depend on the dose and the animal under study. Additionally, in vitro studies using uterine tissue from pregnant and nonpregnant female rats, guinea pigs, rhesus monkeys, cats, and dogs have shown that 20 and 200 µL/mL of aqueous crude extract and 40 µg/mL of hexanoic extract of *M. tomentosa*, as well as 20 µg/mL of kauradienoic acid, significantly increased the number and frequency of contractions in uterine tissue from both pregnant and nonpregnant females [33,47]. Interestingly, the same treatments did not produce significant effects on uterine tissue from rhesus monkey or hamster females [33,47]. Similarly to the administration of *M. tomentosa*, that of *M. frutescens* extract in vitro significantly increased the spontaneous contractility pattern in the uterine tissue of guinea pigs. This effect also occurs in rats independently of any stage of the ovarian cycle or after oophorectomy. Interestingly, a previous administration of sulfate of atropine, a cholinergic antagonist, blocks the effects produced by the *M. frutescens* extract on rat uterine tissue [141], suggesting that this neurotransmitter system participates in the regulation of uterine contraction induced by *Montanoa* extracts.

Furthermore, histological studies on the effects of *Montanoa* extracts on uterine tissue have identified several alterations related to embryo implantation. The instillation of the aqueous crude extract prepared with *M. frutescens* (5 to 50 mg/50 mL) directly in the uterus of 4-day pregnant rats produced a decrease in the number of blastocyst implantation sites [21]. However, 50–100 mg/50 mL of *M. tomentosa* extract had no effects on implantation, but visual clues of reabsorbed embryos, such as abnormal morphology, were identified. In particular, *M. frutescens* also caused significant alterations in uterine structures, such as focal loss of the epithelial lining, thinning of the endometrial surface epithelium, vacuolation and myelin figure formation in cells, thickened blood vessels, and alterations in stromal cells in the rat endometrium [21]. Differences in the effects produced by *M. tomentosa* and *M. frutescens* extracts could be associated with differences in the content of kaurenoic acid in these two plants [142].

Other toxic effects associated with *Montanoa* compounds have been studied on spermatozoa viability. In vitro studies identified that concentrations between 58 and 374 pg/mL of three chemical compounds in *M. frutescens*, i.e., kaurenoic acid, 15-hydroxy-dihydro-kaurenoic acid, and 15-keto-dihydro-kaurenoic acid, were able to inhibit human spermatozoa motility from 50 to 100% compared with the vehicle, in addition to reducing viability by 10% when they were exposed to the compounds for 60 s [75]. These data support that kauradienoic acid and its derivatives may be the active compounds responsible for the toxic effects of *Montanoa* extracts. Additionally, the administration of the aqueous crude extract from *M. tomentosa* for thirty days in young male rats was devoid of effects on androgen-dependent tissues such as the testis, prostate, and seminal vesicles [13]. These results show that the undesired effects produced by *Montanoa* extracts depend on different factors, where the extract dose and sex of the subjects could be involved.

Studies on human females have shown that oral administration of the aqueous crude extract of the leaves of *M. tomentosa* (15 g/per day) on days 18 and 20 of the menstrual cycle produces uterine contractions, cervical dilation, pain, cramps, and vaginal bleeding, similarly to during menstruation. In addition to these effects, an abortive effect was observed following the administration of 1.1 and 1.3 g/kg of the *M. tomentosa* extract for two days to volunteers in early pregnancy, before the surgical interruption of gestation [20,143]. This effect was possibly associated with the significant decrease in plasma progesterone concentrations observed 1 to 2 h after the administration of the extract [143]. Similarly, in pregnant women with dead fetuses, oral administration of 5 and 30 g of the aqueous crude extract of leaves of *M. tomentosa* increased uterine contractility [144], which may eventually contribute to an abortive effect. In contrast, in New Zealand rabbits, 5 mg/day of *M. tomentosa* or *M. frutescens* aqueous crude extract administered from days 3 to 17 of pregnancy did not modify the development of gestation, delivery, or plasma concentration of progesterone [145]. Thus, these results show that the undesired effects of *Montanoa* extracts depend on the organism and the plant species used to prepare the extract.

Finally, in vitro studies have identified that 0.2 to 6 mg/mL of dialyzed-aqueous crude extract from *M. frutescens* produces a hemolytic effect in human erythrocytes. Interestingly, the dialyzed-aqueous crude extract from *M. tomentosa* has not been observed to produce any effect on human blood cells [146]. Together, these data identify the use of plants of the *Montanoa* genus in traditional medicine, considering the possible side and toxic effects that could be observed depending on the characteristics of the subject who consumes it. It is important to highlight that undesired and toxic effects associated with *Montanoa* extracts are controversial and depend on the doses and subjects evaluated. Therefore, it is necessary to evaluate the pharmacological and biological effects of these plants from different perspectives to support or discard the medicinal properties attributed to them in traditional antique medicine.

## 8. Discussion

The World Health Organization (WHO), with 194 member countries, promotes health and safety worldwide with the objective of “the attainment by all peoples of the highest possible level of health” and has recognized “traditional medicine” as a comprehensive term that refers to traditional medicine systems and several forms of indigenous medicine [147]. Traditional medicine includes medication therapies based on the use of herbal medicines, animal parts or their derivatives, and minerals. Additionally, it includes non-medication therapies that do not involve medication, for example, acupuncture and manual and spiritual therapies [147]. The principal strategies of the WHO regarding traditional medicine are focused on three objectives: to develop knowledge of the active management of traditional and complementary medicine; to strengthen quality assurance, security, adequate utilization, and the efficacy of traditional and alternative medicine through the regulation of its products, practices, and professionals; and to promote universal health coverage through the appropriate integration of traditional and complementary medicine services in the provision of health services and self-care [148]. Traditional medicine is widely used, is increasingly and rapidly being adopted in health systems, and is economically important. In developing countries, traditional medicine is used because of historical circumstances and cultural beliefs. Interestingly, in many developed countries, this type of medicine is becoming increasingly popular [149], which is fueled by concerns about potential side effects produced by synthetic chemical drugs used in allopathic medicine.

Additionally, longer life expectancy has increased the risk of developing chronic and debilitating diseases, such as cancer, diabetes, and neurodegenerative and psychiatric disorders. Consequently, patients consider that the use of medicinal plants as traditional complementary medicine offers them gentler and safer treatment options than allopathic medicine [150,151,152,153]. This has led to discussion among WHO members about the need to continue appropriately integrating traditional and complementary medicine services into the provision of health services [149].

The use of medicinal plants in traditional complementary medicine has increased worldwide, not only in rural communities but also in large cities. Unfortunately, many of the plants involved lack preclinical and clinical studies identifying their pharmacological, therapeutic, and toxic effects, which could produce side effects and contribute to the development of other diseases associated with their inadequate use. In this sense, the scientific study of plants and their derivatives is crucial to support or discard their potential use in the health system and strategies to maintain the health of individuals. To confront this great challenge, it is necessary to know the context in which traditional medicine is used, as well as its limitations and restrictions. This information may be found in traditional medicine from ancient cultures around the world and in more recent studies that show the potential medicinal use of several plants and their derivatives. For this reason, the WHO began a virtual dialog with representatives of more than 300 non-governmental organizations about the necessity to extend WHO Traditional Medicine Strategies: 2014–2023 for another two years and vegan developing the new Traditional Medicine Strategy 2025–2030 [149,154] to support evidence and data about policies and practices used in traditional and alternative medicine, as well as global standards and regulations to ensure the safety and quality of the products used in this medicine and support it with scientific, innovative, and technological advances [154] and thereby truly contribute to the maintenance of worldwide human health.

In this narrative review, the study of ancient traditional medicine described in codices such as the *Libellus de Medicinalibus Indorum Herbis* written to reflect the practices of pre-Columbian Mesoamerican cultures allows us to identify the previous uses of *M. tomentosa*, *M. frutescens*, and *M. grandiflora* to treat reproductive disease and anxiety and depression symptoms. Over the years, diverse studies have been conducted on the ethnobotanical, ethnopharmacological, toxicological, and potential medicinal properties of these plants (Table 2), providing structured information from controlled preclinical studies that could support the potential contribution of *Montanoa* genus plants to the future development of phytopharmaceutical products for the treatment of anxiety and depression disorders, among other illnesses.

Current experimental studies have identified the anxiolytic- and antidepressant-like effects of the extracts of three *Montanoa* genus plants: *M. tomentosa*, *M. frutescens*, and *M. grandiflora* (Table 3 and Table 4). These studies have shown that the pharmacological effects of these plants are mediated through interactions with the GABAergic system by activating the GABA_A_/Benzodiazepine receptor complex at different recognition sites of this receptor, which include the recognition site for γ-aminobutyric acid, the binding site for benzodiazepines, and the modulation of the chloride ion channel in the GABA_A_ receptor [17,19]. These mechanisms regulate the inhibitory actions of the GABA_A_ receptor, which are involved in the anxiolytic-like effects of different substances such as approved benzodiazepine drugs, neurosteroids, and polyphenols contained in some plants. It is important to highlight that chronic treatment with benzodiazepines produces pharmacological tolerance that reduces the therapeutic effects over time; in addition, discontinuation of these treatments can produce withdrawal syndrome, which limits the use of these drugs in the long term. Interestingly, at the preclinical level, chronic treatment with *M. tomentosa*, *M. frutescens*, and *M. grandiflora* extracts maintains their anxiolytic- and antidepressant-like effects without producing the withdrawal syndrome [19], which is indicative of the possibility that future Montanoa-derived drugs could be a safer alternative than benzodiazepines in patients who require chronic treatment for anxiety or depression symptoms.

**Figure 4:**
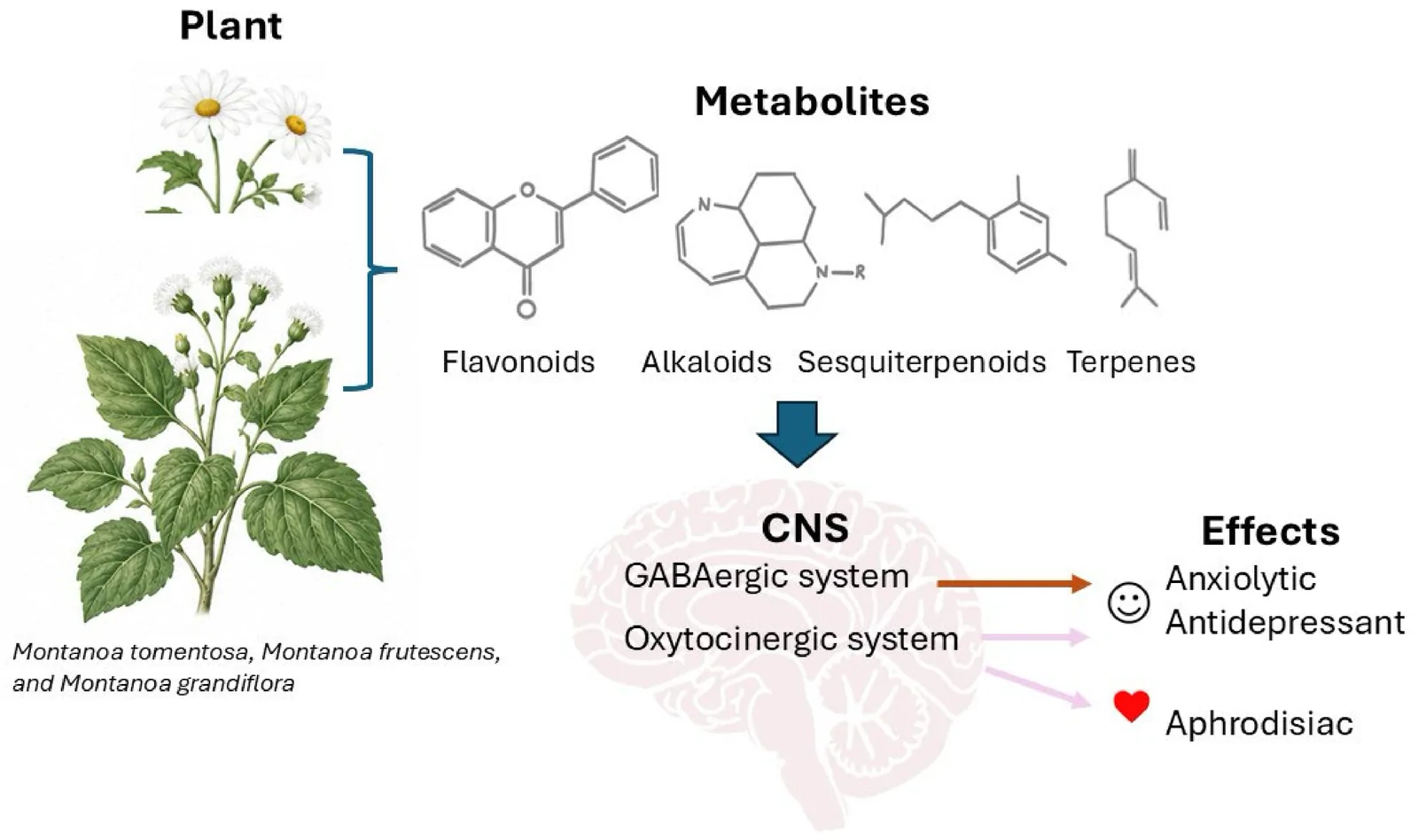
Principal active molecules identified in *M. tomentosa*, *M. frutescens*, and *M. grandiflora* extracts (flavonoids, alkaloids, sesquiterpenoids, and terpenes) and their principal neurochemical system activated (GABAergic and oxytocinergic) to produce anxiolytic- and antidepressant-like effects in preclinical research.

The anxiolytic- and antidepressant-like effects produced by the infusion and extracts of *M. tomentosa*, *M. frutescens*, and *M. grandiflora* are established by the activation of GABA_A_ receptors, as has been shown by the antagonism of the GABA binding site with bicuculine blocking such effects. Similarly, antagonism of the benzodiazepine binding site with flumazenil, or blockage of the chloride ion channel with picrotoxin at the GABA_A_ receptor, also cancels their anxiolytic- and antidepressant-like effects. This highlights the necessity to identify the specific molecules that target the different recognition sites in this receptor, which could be flavonoids, terpenes, or alkaloids (Figure 4), among other molecules identified in these and other plants with anxiolytic- and antidepressant-like effects [155,156,157].

Molecules targeting GABA_A_ receptors—e.g., clinically effective drugs such as benzodiazepines and steroid allopregnanolone—increase chloride ion channel opening, enhance chloride influx, hyperpolarize the neuronal membrane, and exert inhibitory effects related to anxiolytic and antidepressant effects. Furthermore, polyphenolic and terpene molecules, as well as some alkaloids, saponins, and sterols contained in plants, have been reported to produce anxiolytic- and antidepressant effects. Some of these molecules have been identified in the extracts and infusions of the three *Montanoa* plants. Thus, some polyphenols such as genistin, luteolin, kaempferol, and quercetin glycosides, as well as some phenolic compounds such as loliolide, farnesene, phytol, and isochiapin B contained in the extract of *Salvia guaranitica*, produce anxiolytic-like effects [158], among other pharmacological activities. Moreover, the aqueous extract from *Clinopodium pulchellum* contains a high percentage of polyphenols (34.15 mg/g) and flavonoids (29.44 mg/g), which were associated with its anxiolytic- and antidepressant-like effects through the modulation of ion channel targets [159]. In support, a systematic review, meta-analysis, and bibliometric analysis have revealed strong evidence for an anxiolytic-like effect of flavonoids in mice, rats, and zebrafish [160] through actions on different neurotransmission systems, including the serotonergic and GABAergic systems. This is concordant with reports stating that some flavonoids, such as chrysin, isolated from *Passiflora caerulea* and other *Passiflora* species, produce anxiolytic-like effects in rats and zebrafish by targeting the GABA_A_ receptor [161], decreasing the expression of 5HT1_A_ receptors in the dorsal raphe nucleus and increasing 5HT_2A_ receptors in the hippocampus of the rat [162]. Furthermore, this flavonoid has been shown to produce anxiolytic- and antidepressant-like effects in female rats with surgical menopause, depending on the ovarian cycle phase, by targeting the GABA_A_ receptor and increasing Fos expression in lateral septal cells, which also occurs with the phytoestrogen genistein [110,163,164,165]. In summary, diverse polyphenols and flavonoids exert anxiolytic- and antidepressant-like effects by targeting the GABAergic, serotonergic, dopaminergic, and noradrenergic systems, as well as through their antioxidant and anti-inflammatory activity, and by increasing the activation of some neurotrophic factors such as brain-derived neurotrophic factor and nerve growth factor [111,131]. This is important because most clinically effective anxiolytic and antidepressant drugs also involve these effects in their mechanisms of action.

Interestingly, the *M. tomentosa* infusion also activates hypothalamic oxytocinergic neurons [41]. This is an important mechanism of action since recent preclinical and clinical studies have identified that oxytocin may produce anxiolytic and antidepressant effects by modulating emotional and affective states in short lengths of time [166]. It is noteworthy that the activation of hypothalamic oxytocinergic neurons occurs with the acute administration of the *M. tomentosa* extract, and this activation has been associated with motivational behavior, producing aphrodisiac, anxiolytic-, and antidepressant-like effects in preclinical research [15,19]. Interestingly, the extract prepared with *Angelicae sinensis*, *Atractylodes macrocephala*, *Alisma orientale*, and *Ligusticum* chuanxiong, which contains monoterpene glycosides, phenolic compounds, and triterpenes, reduces anxiety and depression-like behavior and improves memory impairment in ovariectomized rats while simultaneously increasing oxytocin concentrations and oxytocin and estrogen receptors in the cortex, as well as the concentrations of glutamate, GABA, serotonin, and noradrenaline in the CA1 hippocampus [167]. The oral administration of *Rosmarinus officinalis* extract reduces anxiety- and depressive-like behavior in male ICR mice, which is associated with a significant increase in central oxytocin levels and the expression of oxytocin receptors, as well as oxytocin protein levels in the limbic system of the mouse brain [168]. The authors suggest that the principal molecules involved in producing these effects are polyphenols such as rosmarinic acid and phenolic diterpenes such as carnosol and carnosic acid. Thus, these molecules may regulate the complex interrelation between oxytocin and some neurotransmitters such as serotonin, dopamine, and noradrenaline, as well as some neurotrophic factors, corticosterone release, and some neuroinflammatory biomarkers [168]. Similarly, the ginsenoside Rg1, a triterpene saponin extracted from *Panax ginseng*, reduces depressive-like behavior in stressed mice, and has been associated with an increase in oxytocin secretion in the hypothalamus [169]. It is important to highlight that lavender oil extracted from *Lavandula angustifolia* or *Lavandula latifolia*, which contains some terpenoid compounds, has been evaluated in preliminary clinical trials. It has been noted to reduce some physiological parameters associated with anxiety such as blood pressure, pulse rate, and cortisol concentration. Moreover, depression and anxiety symptoms have been reduced when lavender oil was administered via inhalation [170]. Interestingly, this same lavender oil has been evaluated in in vitro studies, where it has been observed to increase intracellular Ca^2+^ concentrations in hypothalamic oxytocin neurons [170]. Clinical trials have evaluated the effects of essential oils from different plants, finding that in women, a negative correlation exists between anxiety symptoms and oxytocin levels [171].

Although preclinical studies have identified part of the mechanism of action of *Montanoa* genus plants, which involves the activation of the GABAergic and oxytocinergic systems, it is not possible to discard the participation of other neurotransmission systems, such as serotonergic, noradrenergic, and dopaminergic systems, or the activation of neurotrophic factors such as brain-derived neurotrophic and nerve growth factors, among others. This is because flavonoids, terpenoids, alkaloids, and other secondary metabolites contained in the *Montanoa* plant extracts may activate these neurotrophic factors.

According to preclinical studies described in this review, it is evident that *M. tomentosa* produces the most beneficial effects on anxiety- and depression-like behavior, followed by *M. frutescens* and *M. grandiflora*. Additionally, it is important to highlight a potential limitation in the use of *M. tomentosa* and *M. frutescens* as antidepressant or anxiolytic substances, considering that their therapeutic effects disappear 24 h after treatment withdrawal [19]. Another limitation is noted in the use of high doses of these plants in pregnant women, which may cause uterotonic effects mediated by oxytocin and thus induce abortion, as has been identified in preclinical and clinical research [12,14,32]. In summary, current information regarding the potential anxiolytic- and antidepressant-like properties produced by the three *Montanoa* genus plants supports their traditional use for the treatment of emotional and affective symptoms.

### Future Research Directions to Guide Further Developments in This Field

Future research should focus on conducting multidisciplinary preclinical and clinical studies to identify or discard therapeutic and potential toxic effects associated with a wide range of doses under specific physiological conditions, as well as the sex and age of the experimental subjects. Such an approach will help researchers determine the parameters under which the extracts may produce beneficial health effects without causing potential side effects. Additionally, it is necessary to evaluate the phytochemical profiles and different fractions of the extracts from these three plants to isolate and identify the chemical structure of metabolites responsible for their anxiolytic and antidepressant effects, as well as their potential toxic activity. Equally important are the needs to assess the standardization of products derived from *Montanoa* plants, to identify possible variability in plant-derived compounds, and to evaluate their pharmacokinetic parameters and pharmacological interactions with other drugs to ensure user safety. At present, it is crucial to disseminate knowledge about potential toxic effects to the general population, considering that many people are using these products without any regulation or medical supervision, which could negatively impact health.

Finally, knowledge of the pharmacological properties of the extracts of the three *Montanoa* genus plants discussed in this review comes from preclinical studies performed on laboratory animals, but no clinical trials have been performed. Thus, the results regarding the anxiolytic- and antidepressant-like effects produced by the *Montanoa* extracts require careful consideration, as more specific studies on their pharmacological and neurobiological properties are needed, particularly at the clinical level. Although the lack of translational and clinical research is still a limitation; these results open new perspectives for multidisciplinary studies to contribute to the identification of potential applications in the development of natural alternatives for the treatment of anxiety and depression symptoms in the future.

## 9. Method

### 9.1. Design

This study was designed as a narrative literature review that analyzes information from scientific papers, book chapters, books, historical documents, and official websites to create an informative, critical, and useful synthesis about the topic. As narrative reviews lack systematic methods for identifying, evaluating, and synthetizing information, which could involve bias in how authors include or exclude information to support a position [172], we describe the parameters used to include or exclude studies to ensure an objective inclusion of information. Our study design comprised searching a database, identifying keywords, reviewing abstracts and articles, and documenting the results [173]. Thus, as this paper is a narrative review, PRISMA guidelines were not considered.

### 9.2. Inclusion and Exclusion Criteria of the Bibliographic Material

The inclusion criteria used in the literature search for this review were (1) a focus on *Montanoa* genus plants (*Montanoa tomentosa*, *Montanoa frutescens*, and *Montanoa grandiflora*), under the subjects of ethnobotanical, pre-Columbian Mesoamerican medicine, medicinal properties, chemical compounds, pharmacological properties, toxic effects, mood disorders, and anxiolytic and antidepressant effects; (2) antique medicine described in the *Libellus de medicinalibus indorum herbis*; (3) preclinical and clinical studies on the anxiolytic- and antidepressant-like effects of these three *Montanoa* genus plants; (4) original study articles, book chapters, and books related to the topic; (5) official websites supported by a recognized institution, organization, or scientific society that provide information on the topic; and (6) English and Spanish language articles. The exclusion criteria were as follows: (1) no full text available, only the abstract; (2) incomplete text; (3) unofficial websites; (4) duplicate publication; and (5) theses.

To ensure the inclusion of verifiable evidence, specific criteria were defined for each evidence stream: (a) Ethnobotany: This topic comprised studies reporting traditional or historical uses of *Montanoa* genus plants in sources, e.g., codices such as the *Libellus de medicinalibus indorum herbis*, colonial documents, herbarium records, or peer-reviewed ethnobotanical surveys on human populations of Mesoamerica, while excluding sources lacking verifiable botanical authentication or with no full text available. (b) Phytochemical evidence: This stream included studies reporting on the isolation, structural elucidation, or qualitative identification of secondary metabolites from *M. tomentosa*, *M. frutescens*, or *M. grandiflora*, employing recognized analytical methods (NMR, GC-MS, HPLC, or equivalent); this stream excluded studies on other *Montanoa* species not described in pre-Columbian Mesoamerican codices unless comparative data were directly relevant. (c) Pharmacology: This topic included controlled preclinical studies (in vivo and in vitro) evaluating anxiolytic- or antidepressant-like effects, aphrodisiac properties, reproductive pharmacology, or studies on the mechanism of action; this section excluded studies with no declared doses, without a control group, or that lacked behavioral evaluations. (d) Toxicology: This section included experimental studies reporting adverse, toxic, or negative uterotonic effects of Montanoa extracts or their isolated compounds in any organism or cell system; this section excluded case reports without biochemical confirmation and non-peer-reviewed sources.

### 9.3. Article Search

Data on the topic by the inclusion criteria were searched for and gathered through specialized databases, including PubMed/Medline, Science Direct, Google Scholar, IMBIOMED, and Latindex. The literature search was conducted without considering a specific publication period; this permitted the inclusion of historical sources up to current publications (30 May 2026). The database-specific search strings employed are the following: PubMed/Medline: “Montanoa” [MeSH Terms] OR “*Montanoa tomentosa*” OR “*Montanoa frutescens*” OR “*Montanoa grandiflora*” in [Title/Abstract] AND “ethnopharmacology” OR “ethnobotany” OR “phytochemical” OR “pharmacology” OR “anxiolytic” OR “antidepressant” OR “toxic effect” OR “side effect” in [Title/Abstract]. ScienceDirect [Title/Abstract]: “Montanoa” AND “anxiolytic” OR “antidepressant” OR “phytochemical” OR “ethnobotany” OR “toxicology”. Google Scholar [Title/Abstract]: “*Montanoa tomentosa*” OR “*Montanoa frutescens*” OR “*Montanoa grandiflora*” combined with “ethnobotany” OR “phytochemical” OR “pharmacology” OR “anxiolytic” OR “antidepressant” OR “toxic effect” OR “side effect”. IMBIOMED and Latindex: “Montanoa” AND “ansiolítico” OR “antidepresivo” OR “fitoquímica” OR “etnobotánica” OR “toxicología”. Additionally, the Virtual Library and the Library and Information Services Unit (USBI-Xalapa) of Universidad Veracruzana, México: https://www.uv.mx/bvirtual/ (accessed on 1 May 2026) and https://catbiblio.uv.mx/ (accessed on 10 May 2026), were used to search for physical documents not available online using the same keyword combinations in both Spanish and English.

## 10. Conclusions

Preclinical studies have identified that *M. tomentosa*, *M. frutescens*, and *M. grandiflora* extracts reduce specific behaviors related to anxiety and depression states at the experimental level in rats. These effects are regulated through action on the GABA_A_ receptor and oxytocinergic systems, without excluding the participation of other neurotransmission systems and neurotrophic factors that have not yet been studied. Current preclinical data suggest that *M. tomentosa* extracts could exhibit the most significant effects on anxiety- and depressive-like behaviors, considering the low doses required to produce these effects, followed by *M. frutescens* and *M. grandiflora*, which require higher doses. However, we need to be cautious, considering that *M. frutescens* and *M. grandiflora* have been less studied with respect to their pharmacological and toxic effects, phytochemical profile, and anxiolytic and antidepressant properties. It appears that their effects could be produced by secondary metabolites such as flavonoids, alkaloids, and some terpenes. However, despite promising results, the potential therapeutic effect of these three plants for the treatment of anxiety- and depression-like symptoms has not been sufficiently evaluated with respect to their toxicity, the standardization of products, the identification of possible variability in plant-derived compounds, or their pharmacokinetic parameters and pharmacological interaction with other drugs. This is a great challenge for future studies, which are required to obtain sufficiently robust preclinical evidence on the safety and efficacy of *M. tomentosa*, *M. frutescens*, and *M. grandiflora* to subsequently conduct translational studies and all phases of clinical trials to ensure the therapeutic properties of these plants for the treatment of psychiatric disorders in humans.

## Figures and Tables

**Table 1:** Species, subspecies, and varieties of the *Montanoa* genus distributed in America *.

Genus	*Montanoa*
Species	*andersonii*, *aschenbornii*, *atriplicifolia*, *auriculata*, *bipinnatifida*, *creanata*, *echinaceae*, *frutescens*, *gracilis*, *grandiflora*, *guatemalensis*, *hibiscifolia*, *imbricata*, *josei*, *karwinskii*, *laskowskii*, *lehmannii*, *leucantha*, *liebmannii*, *mollissima*, *moritziana*, *olivae*, *ovalifolia*, *pteropoda*, *quadrangularis*, *reveallii*, *rosei*, *speciosa*, *standleyi*, *ternifolia*, *tomentosa*, *triloba*, and *xanthiifolia*
Subspecies	*Montanoa leucantha arborescens*, *Montanoa leucantha leucantha*, *Montanoa tomentosa rosei*, and *Montanoa tomentosa tomentosa*
Varieties	*Montanoa tomentosa microcephala*, *Montanoa tomentosa xanthifolia*, and *Montanoa tomentosa xanthiifolia*

* Based on the SEINet Portal Network, 2022 [37].

**Table 2:** Summary of the ethnobotanical, phytochemical, pharmacological, and toxicological data from *M. tomentosa*, *M. frutescens*, and *M. grandiflora*. These plants have been used in the traditional medicine of pre-Columbian Mesoamerican cultures since before the 16th century, mainly for treating women’s illnesses.

Field of Study	*M. tomentosa*	*M. frutescens*	*M. grandiflora*	Ref.
Ethnobotanical	The infusion of the aerial parts is used as a contraceptive agent, inducer of uterine contractions and labor, analgesic, and regulator of menstrual cycle, aphrodisiac agent, as well as for the treatment of anxiety and depression.	The infusion of the aerial parts is used to treat anxiety and mood disorders, used as a labor inducer, analgesic, and regulator of the menstrual cycle.	The infusion from leaves and bark is used to ameliorate toothache, as an analgesic and anti-inflammatory, and to treat “anxious states”.	[12,13,14,23,24,25,29,32]
Major phytochemical constituents of three *Montanoa* genus plants and crude extracts	Diterpenes: kaurenoic acid, grandiflorenic acid, and monoginoic acid. Sesquiterpene lactones: zoapatanolides A and B and guaiamolide compounds such as zoapatanolides C and D. Borneol acetate, β-cubebene, and β-caryophyllene.	The aqueous crude extract contains flavonoids, alkaloids, sesquiterpenoids, and terpenes.	Flavonoids, alkaloids, sesquiterpenoids, terpenes, and sterols.	[19,49,50,51,52,53,54,55,56,60]
Hexane (H)/Chloroform I	H: kaouradienoic acid.	H: Kaurenoic acid, 15α-isovalerate, 15α-hydroxy-dihydroxy kaurenoic acid, and methyl ester of 15-keto-dihydro-kaurenoic acid.	C: sesquiterpene lactone 6β-hydroxy-germacradien-8,12-olide, caryophyllene oxide, and triterpene lup-20(29)-en-3-ol acetate.	[19,51,52,53,54,55,56]
Petroleum	Sesquiterpene lactones: zoapatanolides A, C, and D and pumilin. Diterpenes: zoapatols A and B, guaianolide, zoapatanolide F, zoapatanol, and montanol.	Caryofillene, taraxasterol acetate, kaurenic acid, and 15α-isovaleriate.	Unexplored.	[19,49,50,51,52,53,54,55,56]
Essential oil	Borneol acetate, β-cubebene, and β-caryophyllene.	Unexplored.	α- and β-pinene, limonene, menthol, eucalyptol, terpineol, geranyl acetate, terpenil acetate, citronellal, thymol, α-phellandrene, camphene, guaiacol, methyl eugenol, and manol.	[19,49,50,51,52,53,54,55,56]
Infusion	Flavonoids: nicotiflorin and isoquercitrin; sesquiterpenoid oxepane; 21-normontanol; and zoapatanolides A, C, and D. Monoterpenes: sabinene, α-pinene, α-thujene, and α-gurjunene. Sesquiterpenes: caryophyllene and germacrene D. Terpenoids: tomentanol kaurene diterpenes: kaur-16-en-19-oic acid, kaur-9(11)16-dien-19-oic acid, angeloylgrandifloric acid, monoginoic acid, and grandifloric acid.	Unexplored.	Unexplored.	[19,49,50,51,52,53,54,55,56]
Others	Methylene chloride extract: zoapatanol; sesquiterpenoids: pretomentol, pretomentol acetate, prezoapatanol, and pretomexanthol. The root extract contains monoginoic acid, kaurenoic acid, and oxepane 3-C-methyl-α-D-allofuranose, a 2-zoapatanol analog. Alcoholic, organic solvent, and aqueous extracts: zoapatanol, montanol, nicotiflorin, isoquercitrin, tomexanthin, tomentol, zoapatanolide A, kauradienoic acid, grandiflorenic acid, and methyl grandiflorenate.	Trichloremethane extract: montafrusin and montafrusin acetate. Different extracts contain sesquiterpenoids: montafrusin and montafrusins A and B. Diterpenes such as kaurenoic acid and eudesmanolides: montafrusins C, D, E, and F.	Unexplored.	[19,49,50,51,52,53,54,55,56,65]
Reported pharmacological activity	Aphrodisiac effects in male rats through the activation of the oxytocinergic system. Anxiolytic-like effects through the activation of GABA_A_ receptors, which were blocked by the non-competitive antagonist picrotoxin.	Aphrodisiac effects in male rats through oxytocinergic neurons. Anxiolytic-like effects comparable to diazepam in males and female rats during the metestrus–diestrus phases. Antidepressant-like effects in male rats in the FST.	Increases sexual behavior in male rats. Anxiolytic-like effects in male and female rats during metestrus–diestrus phases. Immediate antidepressant-like effects in male rats in the FST.	[15,16,17,18,32,40,44,46,58,65,107]
Main mechanisms of action involved in their pharmacological effects	The anxiolytic effect is associated with binding to various sites of the GABA_A_/benzodiazepine receptor complex; this effect is blocked by the antagonist picrotoxin. The antidepressant effect is associated with the activation of oxytocin cells in the paraventricular and supraoptic nuclei.	Action on the GABA_A_ receptor, blocked by picrotoxin. The immediate antidepressant effect suggests a mechanism associated with GABA_A_ receptors and not associated with the monoaminergic system like fluoxetine.	Activates oxytocin cells. Acts on the GABA_A_ receptor, blocked by picrotoxin; the mechanisms of its antidepressant-like effect have not been explored beyond GABAergic participation.	[15,16,17,18,19,41]
Toxicological	Abortive effects in human and rhesus monkeys by increasing uterine contraction, likely by oxytocinergic activity. In female guinea pigs and cats, they increase blood pressure and uterotonic activity, leading to abortive effects. The hexanoic extract increases uterine contractions related to abortive effects in females.	Increases contractility of uterine tissue in guinea pigs. Decreases the number of blastocyst implantation sites and loss of the epithelial lining, thinning of the endometrial surface epithelium, vacuolation and myelin figure formation in cells, thickened blood vessels, and alterations in stromal cells in rat endometrium. Inhibits spermatozoa motility and produces hemolytic effects on erythrocytes in humans.	No significant toxic effects have been reported.	[13,21,32,62,75,146]
Level of experimental evidence	Preclinical and clinical evidence. Anxiolytic, antidepressant, and aphrodisiac effects have been studied only in preclinical research on the Wistar rats. No clinical trials have been performed to date. Uterotonic and abortive effects have been studied at the preclinical level in rats, hamsters, guinea pigs, cats, and rhesus monkeys. A few human studies at the clinical level have studied the uterotonic and abortive effects in small non-randomized pilot studies.	Preclinical evidence. Anxiolytic, antidepressant, and aphrodisiac effects have been studied only at preclinical level in Wistar rats. Reproductive toxicity has been assessed in guinea pigs and rats. In vitro assays have been conducted to evaluate toxicity in human erythrocytes and spermatozoa.	Preclinical evidence. This species has been studied the least. In vivo studies have identified the anxiolytic- and antidepressant-like effects of their aqueous crude extracts and infusions via preliminary phytochemical analysis. The administration of extract infusion has not produced toxic effects in preclinical research on rats. Moreover, no long-term toxicological studies or clinical trials have been performed.	[12,13,15,18,19,20,21,32,40,41,47,75,107,140,141,142,146]

**Table 3:** Preclinical studies that have explored the anxiolytic-like effects of *Montanoa tomentosa*, *Montanoa frutescens*, and *Montanoa grandiflora*.

Plant	Subjects/Extract Type	Doses/Administration Route/Treatment duration	Model Used	Results/Effect	Potential Mechanism of Action	Ref.
*Montanoa tomentosa*	Adult male Wistar rats/freeze-dried leaves.	1.5, 3, 6, and 12 mg/kg, p.o., single dose, 30 min before behavioral tests.	BBT	Doses of 3, 6, and 12 mg/kg significantly reduced cumulative burying time and increased probe exploration, while those of 6 and 12 mg/kg significantly increased burying behavior latency, supporting an anxiolytic-like effect. This effect was similar to that produced with 0.5 and 1 mg/kg of muscimol.	The anxiolytic-like effects identified in the three behavioral tests were prevented by a prior i.p. injection of the picrotoxin, bicuculine, and flumazenil antagonists of GABA_A_ receptors. This supports the involvement of the GABAergic system in the anxiolytic-like effects of this plant.	[17]
			EPM	Only the dose of 3 mg/kg significantly increased time spent in and the percentage of entries to open arms, which supports an anxiolytic-like effect. This effect was similar to that produced with 0.25 mg/kg of muscimol.		
			HBT	Only the dose of 3 mg/kg significantly increased the number of head dips, which is related with an anxiolytic-like effect.		
			LAT	None of the evaluated doses produced significant effects on motor activity.		
			RRT	Only the dose of 12 mg/kg increased the number of falls, which could be associated with sedation.		
*Montanoa tomentosa*	Adult female Wistar rats considering the estrus cycle/freeze-dried leaves.	0.38, 0.75, 1.5, and 3 mg/kg, p.o., single dose, 60 min before behavioral tests.	EPM	Doses of 1.5 and 3 mg/kg significantly increased the percentage of time spent in and the number of entries to open arms, which supports an anxiolytic-like effect similar to that with 2 mg/kg of diazepam.	The anxiolytic-like effect produced with 1.5 mg/kg was blocked by a previous i.p. injection of the picrotoxin antagonist of GABA_A_ receptors. This supports the involvement of the GABAergic system in the anxiolytic-like effects of this plant in female rats.	[107]
			LAT	No treatment effect was detected.		
	Adult female Wistar rats 3 weeks post ovariectomy considering the estrus cycle phases/freeze-dried leaves	0.38, 0.75, 1.5, and 30 mg/kg, p.o., single dose, 60 min before behavioral tests.	EPM	Only the dose of 1.5 mg/kg significantly increased the percentage of time spent in and the number of entries to open arms, which suggests there is an anxiolytic-like effect similar to that of diazepam use.		
			LAT	Doses of 0.38, 0.75, and 1.5 mg/kg significantly increased locomotor activity.		
	Adult female Wistar rats 3 weeks post ovariectomy under 2 mg/kg progesterone withdrawal challenge/freeze-dried leaves.	0.38, 0.75, 1.5, and 30 mg/kg, p.o., single dose, 60 min before behavioral tests.	EPM	Only doses of 0.38 and 0.75 mg/kg significantly increased the percentage of time spent in and the number of entries to open arms, which suggest an anxiolytic-like effect similar to that with 2 mg/kg of diazepam.		
			LAT	No treatment effects were detected.		
*Montanoa tomentosa*	Adult female Wistar rats 12 weeks post ovariectomy/aqueous crude extract of aerial parts.	25, 50, and 75, mg/kg, p.o., single dose, 30 min before behavioral tests.	EPM	Only doses of 50 and 75 mg/kg significantly increased the percentage of time spent in and the number of entries to open arms, which suggests there is an anxiolytic-like effect similar to that with 2 mg/kg of diazepam.	Not explored.	[40]
			LAT	No significant changes were detected in crossing and rearing, while the dose of 75 mg/kg increased grooming behavior.		
*Montanoa frutescens*	Adult male Wistar rats/aqueous crude extract of aerial parts.	25, 50, and 75, mg/kg, p.o., single dose, 30 min before behavioral tests.	EPM	Only the dose of 50 mg/kg significantly increased the percentage of time spent in and the number of entries to open arms, which suggests there is an anxiolytic-like effect similar to that with 2 mg/kg of diazepam.	The anxiolytic-like effect produced with 50 mg/kg was blocked by a previous i.p. injection of the picrotoxin antagonist of GABA_A_ receptors. This supports the involvement of the GABAergic system in the anxiolytic-like effects of this plant.	[15]
			LAT	Doses of 25 and 50 mg/kg did not produce significant effects, while a dose of 75 mg/kg produced hypoactivity similar to that with 4 mg/kg of diazepam.		
*Montanoa frutescens*	Adult female Wistar rats considering the estrus cycle phases/aqueous crude extract of aerial parts.	25 and 50 mg/kg, p.o., single dose 30 min before behavioral tests.	EPM	Both doses significantly increased the percentage of time spent in and the number of entries to open arms during metestrus–diestrus phases, similar to that with 2 mg/kg of diazepam. This suggests an anxiolytic-like effect. No treatment effects were detected during proestrus–estrus phases.	Not explored.	[18]
			LAT	No significant effects were detected in this test.		
*Montanoa grandiflora*	Adult female Wistar rats considering the estrus cycle phases/aqueous crude extract of aerial parts.	25 and 50 mg/kg, p.o., single dose, 30 min before behavioral tests.	EPM	Only a dose of 50 mg/kg significantly increased the percentage of time spent in and number of entries to open arms during metestrus–diestrus phases, similar to that with 2 mg/kg of diazepam, this suggests there is an anxiolytic-like effect. No treatment effects were detected during proestrus–estrus phases.	Not explored.	[18]

p.o, oral route; i.p., intraperitoneal injection; HBT, hole-board test; BBT, burying behavioral test; EPM, elevated plus maze; LAT, locomotor activity test; RRT, Rota-Rod Test.

**Table 4:** Preclinical studies that have explored the antidepressant-like effects of *Montanoa tomentosa*, *Montanoa frutescens*, and *Montanoa grandiflora*.

Plant	Subjects/Extract Type	Doses/Administration Route/Treatment Duration	Model Used	Results/Effect	Potential Mechanism of Action	Ref.
*Montanoa tomentosa*	Adult male Wistar rats/infusion of the plant’s aerial parts.	50 mg/kg, p.o., single dose, 30 min before behavioral tests.	FST	This dose significantly increases the latency to the first immobility and reduces the total time of immobility in FST, suggesting a moderate antidepressant-like effect compared with 10 mg/kg of fluoxetine antidepressant.	The infusion, but not fluoxetine, significantly increased the number of Fos/Oxytocin cells in the PVN and SON, which suggests that the oxytocinergic system participates in the antidepressant-like effects of this plant.	[41]
			LAT	The evaluated doses did not produce significant effects on motor activity.		
*Montanoa tomentosa*	Adult male Wistar rats/infusion of the plant’s aerial parts.	50 mg/kg, p.o., 28 consecutive days of treatment. The effect was evaluated 30 min after the last treatment.	FST	This dose significantly reduces the total time of immobility in FST, which supports an antidepressant-like effect similar to that produced with 1 mg/kg of fluoxetine antidepressant.	None of the treatments chronically administered produced significant effects on the number of Fos/Oxytocin cells in the PVN and SON. This only suggests that the serotonergic or GABAergic system could be involved in the antidepressant-like effect of this plant.	[139]
			LAT	None of the treatments produced significant effects on motor activity.		
*Montanoa frutescens*	Adult male Wistar rats/infusion of the plant’s aerial parts.	25 and 50 mg/kg, p.o., 28 consecutive days of treatment. The effect was evaluated 60 min after treatment administration on days 1, 7, 14, 21, and 28 and 24 and 28 h after withdrawal.	FST	Only the dose of 50 mg/kg significantly reduced the total time of immobility from days 1 to 28 of treatment, while fluoxetine and Remotive antidepressants required 14 days of treatment to reduce this variable. The effect of Montanoa extract disappeared 24 h after withdrawal, which was not observed with the other treatments.	The antidepressant-like effect produced by the extract was blocked by a previous i.p. injection of the picrotoxin antagonist of the GABA_A_ receptors. This suggests that the GABAergic system is involved in the antidepressant-like effects of this plant.	[19]
			LAT	No significant effect on motor activity was produced by these treatments.		
*Montanoa grandiflora*	Adult male Wistar rats/infusion of the plant’s aerial parts.	25 and 50 mg/kg, p.o., 28 consecutive days of treatment. The effect was evaluated 60 min after treatment administration on days 1, 7, 14, 21, and 28 and 24 and 28 h after withdrawal.	FST	Only the dose of 50 mg/kg significantly reduced the total time of immobility from days 1 to 28 of treatment, while fluoxetine and Remotive antidepressants required 14 days of treatment to reduce this variable. The effect of Montanoa extract disappeared 24 h after withdrawal, which was not observed with fluoxetine and Remotive.	The antidepressant-like effect produced by the extract was blocked by a previous i.p. injection of the picrotoxin antagonist of the GABA_A_ receptors. This suggests that the GABAergic system is involved in the antidepressant-like effects of this plant.	[19]
			LAT	No significant effect on motor activity was produced by the treatments.		

p.o, oral route; i.p., intraperitoneal injection; FST, forced swim test; LAT, locomotor activity test; PVN, paraventricular nucleus of the hypothalamus; SON, supraoptic nucleus of the hypothalamus.

## Data Availability

No new data were created or analyzed in this study. Data sharing is not applicable to this article.

## Abbreviations

The following abbreviations are used in this manuscript:

GABA_A_	γ-aminobutyric acid type A receptor
GABA	γ-aminobutyric acid
WHO	World Health Organization
PVN	Paraventricular nucleus of hypothalamus
p.o	Oral rout
i.p.	Intraperitoneal injection
HBT	Hole-Board Test
BBT	Burying Behavioral Test
EPM	Elevated Plus Maze
LAT	Locomotor Activity Test
RRT	Rota-Rod Test
FST	Forced Swim Test
SON	Supraoptic Nucleus of the hypothalamus
SIREI	Sistema de registro y evaluación de la Investigación
